# Patterns of enrichment and acceleration in evolutionary rates of promoters suggest a role of regulatory regions in cetacean gigantism

**DOI:** 10.1186/s12862-023-02171-5

**Published:** 2023-10-24

**Authors:** Felipe A. Silva, Agnello C. R. Picorelli, Giovanna S. Veiga, Mariana F. Nery

**Affiliations:** Dept of Genetics, Evolution, Microbiology & Immunology, Institute of Biology, University of Campinas, Rua Monteiro Lobato, 255, Campinas, 13083-862 SP Brazil

**Keywords:** Cetaceans, Gigantism, Molecular evolution, Regulatory regions, Promoter

## Abstract

**Background:**

Cetaceans (whales, porpoises, and dolphins) are a lineage of aquatic mammals from which some species became giants. Only recently, gigantism has been investigated from the molecular point of view. Studies focused mainly on coding regions, and no data on the influence of regulatory regions on gigantism in this group was available. Accordingly, we investigated the molecular evolution of non-coding regulatory regions of genes already described in the literature for association with size in mammals, focusing mainly on the promoter regions. For this, we used Ciiider and phyloP tools. Ciiider identifies significantly enriched transcription factor binding sites, and phyloP estimates the molecular evolution rate of the promoter.

**Results:**

We found evidence of enrichment of transcription binding factors related to large body size, with distinct patterns between giant and non-giant cetaceans in the *IGFBP7* and *NCAPG* promoters, in which repressive agents are present in small cetaceans and those that stimulate transcription, in giant cetaceans. In addition, we found evidence of acceleration in the *IGF2*, *IGFBP2*, *IGFBP7*, and *ZFAT* promoters.

**Conclusion:**

Our results indicate that regulatory regions may also influence cetaceans’ body size, providing candidate genes for future research to understand the molecular basis of the largest living animals.

**Supplementary Information:**

The online version contains supplementary material available at 10.1186/s12862-023-02171-5.

## Background

Cetaceans (whales, porpoises, and dolphins) compose a lineage exclusively of aquatic mammals, classified into two groups: the odontocetes—animals with teeth—and mysticetes—animals with baleen plates that allow for food filtration [1]. Cetaceans evolved from small-sized terrestrial ancestors approximately 50 Myr ago, during the Eocene [2]. By then, cetaceans started recolonizing the aquatic environment, a process followed by extensive morphological and physiological modifications such as reducing olfactory and gustatory systems, loss of hind limbs, and modifications toward a hydrodynamic body [3]. Some cetacean species have become gigantic, with colossal measures that are not achieved by living animals. Gigantism results from species evolving enormous body sizes compared with their small-sized ancestors. This feature affects critical life-history traits, such as fecundity, due to the consequent lower reproductive rate and an overall reduction in effective population genetic size (N_e_) due to lower population densities [4, 5]. Despite this, some cetacean species reach large body sizes that are unique among living animals, ranging from the impressive gray whale (*Eschrichtius robustus*) with 15 m to the colossal blue whale (*Balaenoptera musculus*) that reaches up to 30 m [6, 7].

Some ecological hypotheses have been proposed to explain the large body proportions in cetaceans, such as thermoregulation [8], a wider space available in the aquatic environment to explore new niches [9], and also food acquisition, which in mysticetes is associated with filtration of small prey [10], and in sperm whale, the largest odontocete at 20 m in length [11], with the ability to dive to extraordinary depths to capture their prey [12]. In addition to these ecological causes, the genetics behind body size has been recently investigated, taking advantage of the sequenced cetacean genomes. For example, evolutionary analyses have shown signatures of positive selection on size-related genes in cetaceans. Sun et al. 2019 [13] found evidence of selection in genes related to small size in cetaceans, such as ACAN, OBSL1, and GRB10 genes; whereas, in giant cetaceans, genes possibly evolving under positive selection were those with known roles in promoting growth and large sizes, such as CBS, EIF2AK3, and PLOD1 genes. Still, these studies focused only on coding regions, and information on the influence of regulatory regions on gigantism in this group is scarce.

Non-coding sequences with regulatory functions (e.g., promoters and enhancers) coordinate the spatial-temporal expression of genes [14]. Although regulatory regions are not under the same constraints as coding sequences, highly conserved sequence blocks in different species indicate evolutionarily conserved functions [15, 16]. On the other hand, modifications of gene regulatory elements have been associated with phenotypic changes in animal evolution, such as pigmentation changes in dogs [17], bristle patterns in flies [18], and skeletal differences in fish [19]. This approach to studying transcription factors is currently facilitated by computational methods that can identify potential candidate gene regulatory elements by detecting regions of the genome that exhibit evolutionary conservation or acceleration [16].

**Fig. 1:**
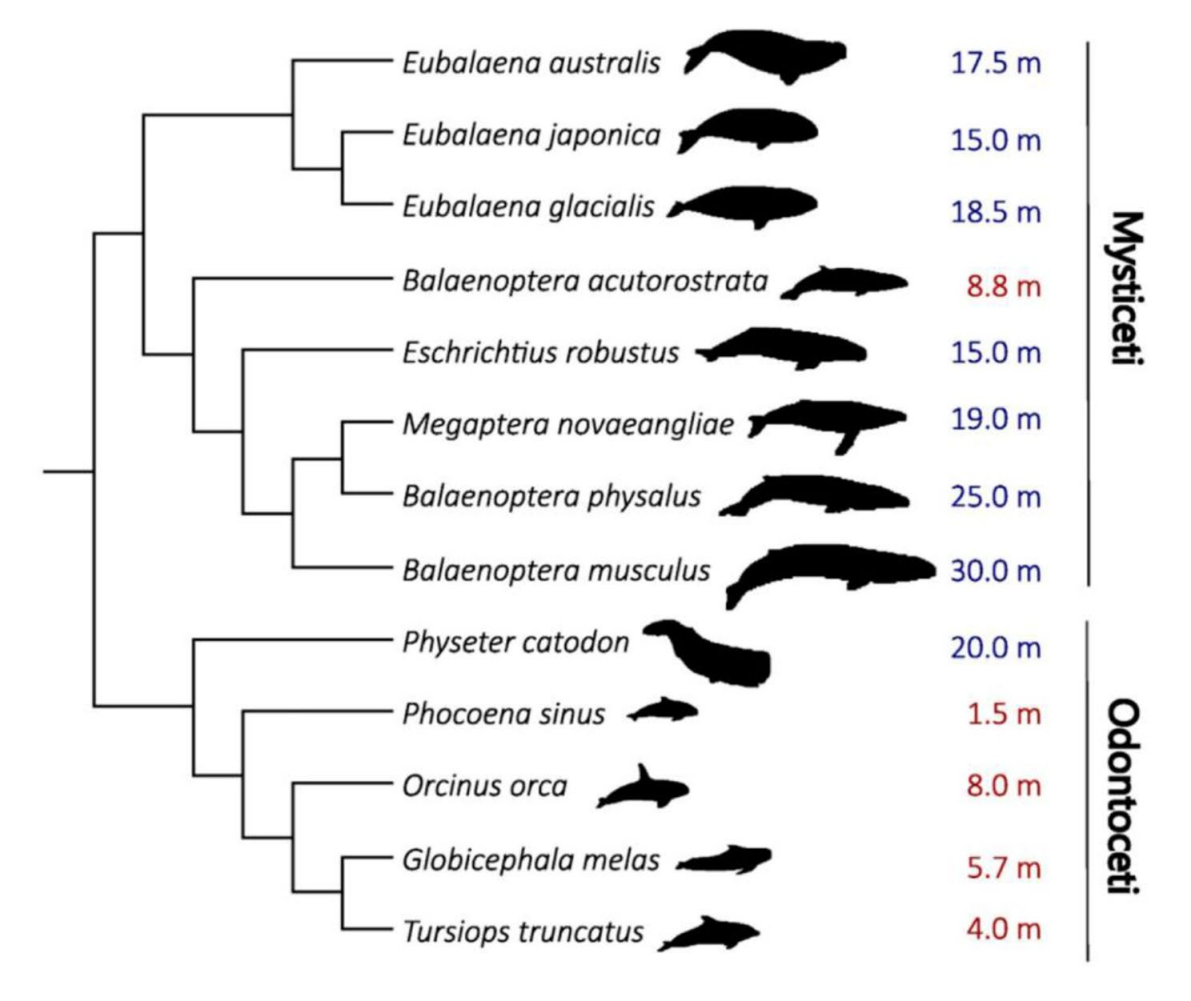
The adult average size, in meters, of all cetacean species included in this study. Blue values indicate giant cetaceans, and red values non-giant cetaceans. Gigantism in this group is defined by body measurements with an average length of 10 m. *Physeter catodon*, *Eschrichtius robustus*, *Eubalaena japonica*, *Eubalaena australis*, *Eubalaena glacialis*, *Megaptera novaeangliae*, *Balaenoptera physalus*, and *Balaenoptera musculus* are classified as giants. Size values are from the “Encyclopedia of Marine Mammals” and the phylogeny from McGowen et al., 2020 [20]

Comparative genome-wide regulatory sequence approaches can provide insights into the evolutionary history of large body size in cetaceans. Specifically, our study focuses on species that are at least 10 m long and classified as giants. In Fig. 1 we present the cetacean species included in our investigation, highlighted in blue for giants and red for non-giants, based on their average size. Accordingly, we investigated the molecular evolution of non-coding regulatory regions of genes previously described in the literature as being associated with size in mammals, such as EGF, *GHSR*, *IGF2*, *IGFBP2*, *IGFBP7*, *LCORL*, *NCAPG*, *PLAG1*, and *ZFAT*, focusing on cetaceans. Our analyses were performed within a phylogenetic framework, where each promoter contained a consistent set of 52 species, including 39 from different orders of mammals and 13 species of cetaceans, of which eight were classified as giants with a minimum length of 10 m. The objective was to investigate differences in the enrichment of Transcription Factor Binding Sites (TFBS) between giant and non-giant cetaceans, as well as to identify potential evolutionary acceleration in these animals with large body sizes.

## Results

### Phylogenetic reconstruction

To identify potential candidate genes that may contribute to gigantism in cetaceans, we examined the evolution of their regulatory regions. To this end, we generated phylogenetic trees for each gene selected in this study using a maximum likelihood approach. These trees were constructed to visually explore the evolutionary relationships among the promoter sequences of the species included in our dataset, focusing on potential convergence among giant species. Specifically, we generated phylogenetic trees for the promoter region (-1500 bp to + 500 bp from TSS) of each gene. The promoter regions were defined as the sequences upstream of the transcription start site, as this is where regulatory elements, such as transcription factor binding sites, are typically located. The correct grouping of mysticetes and odontocetes was observed in most of the phylogenetic trees, along with other groups of mammals, such as Artiodactyla, Carnivora, Primate, Cingulata, and Chiroptera (Additional file 1: Supplementary Figures S1-S8 show the phylogenetic trees for each promoter).

**Fig. 2:**
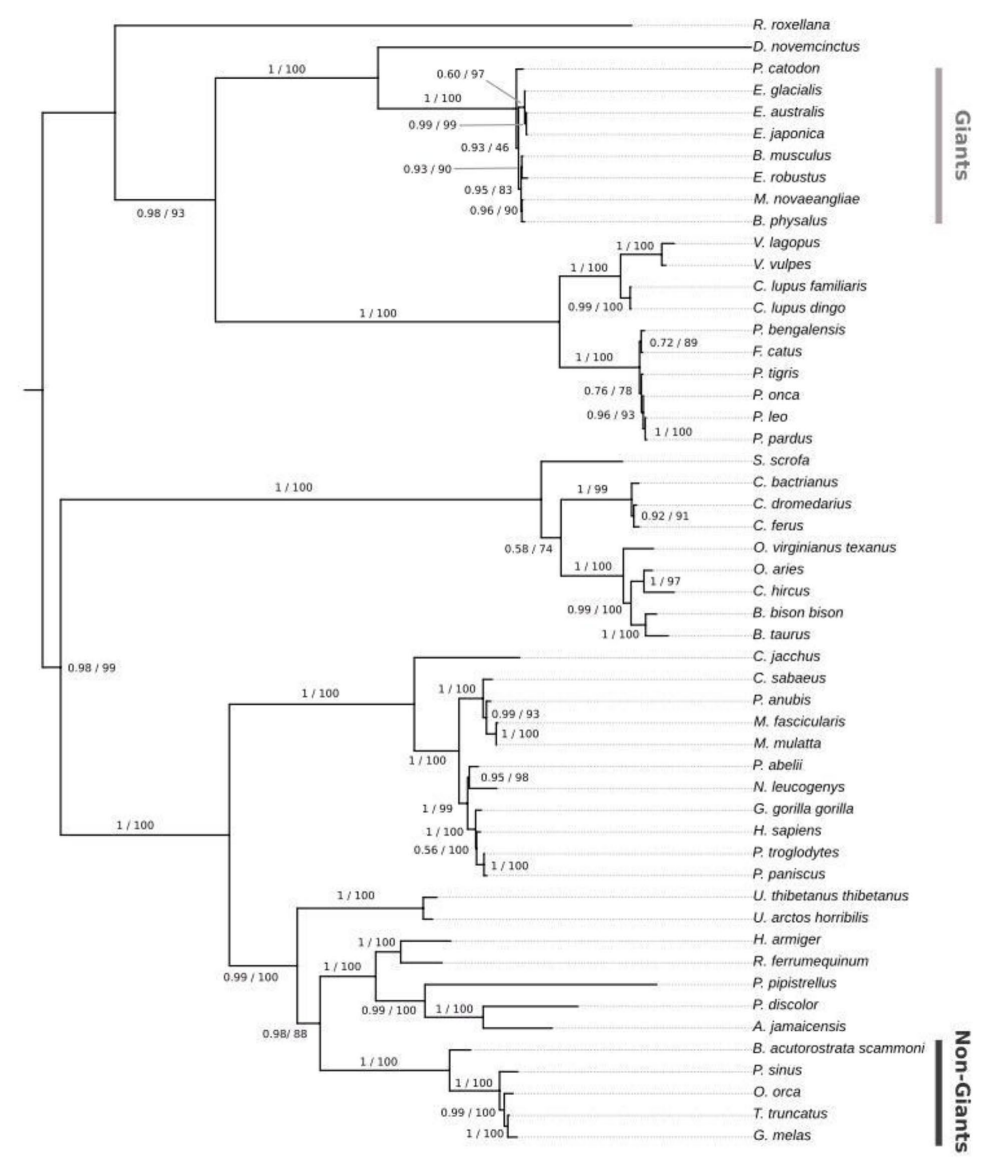
The formation of a clade with only giant cetaceans (the sperm whale odontocete and the other large mysticetes) and another with smaller cetaceans (the mysticete minke whale [*Balaenoptera acutorostrata*] alongside the remaining odontocetes) in both Maximum likelihood tree generated by IQ − TREE and Bayesian tree generated by Mr Bayes v3.2.6 constructed from the promoter region of the NCAPG gene. Numbers under nodes represent bootstrap support (right) and Bayesian posterior probability (left)

One exception was observed in the NCAPG promoter, in which the odontocete sperm whale (*Physeter catodon*) was grouped within the mysticete clade, and the mysticete minke whale (*Balaenoptera acutorostrata*) was grouped with the odontocetes (Fig. 2). Thus, the NCAPG tree had a clade formed by the gigantic animals included in this dataset, *Balaenoptera musculus, Physeter catodon, Eschrichtius robustus, Megaptera novaeangliae, Balaenoptera physalus, Eubalaena australis*, *Eubalaena glacialis*, and *Eubalaena japonica*. To confirm this scenario, we performed a Bayesian approach, which returned the same grouping by size presented previously. This may be due to factors such as evolutionary convergence or rapid evolution of regulatory elements in this particular gene. To explore this further, we performed additional analyses to investigate the rate of evolution and the dynamics of changes in the TFBS of the promoters.

### Regulatory regions analyses

**Fig. 3:**
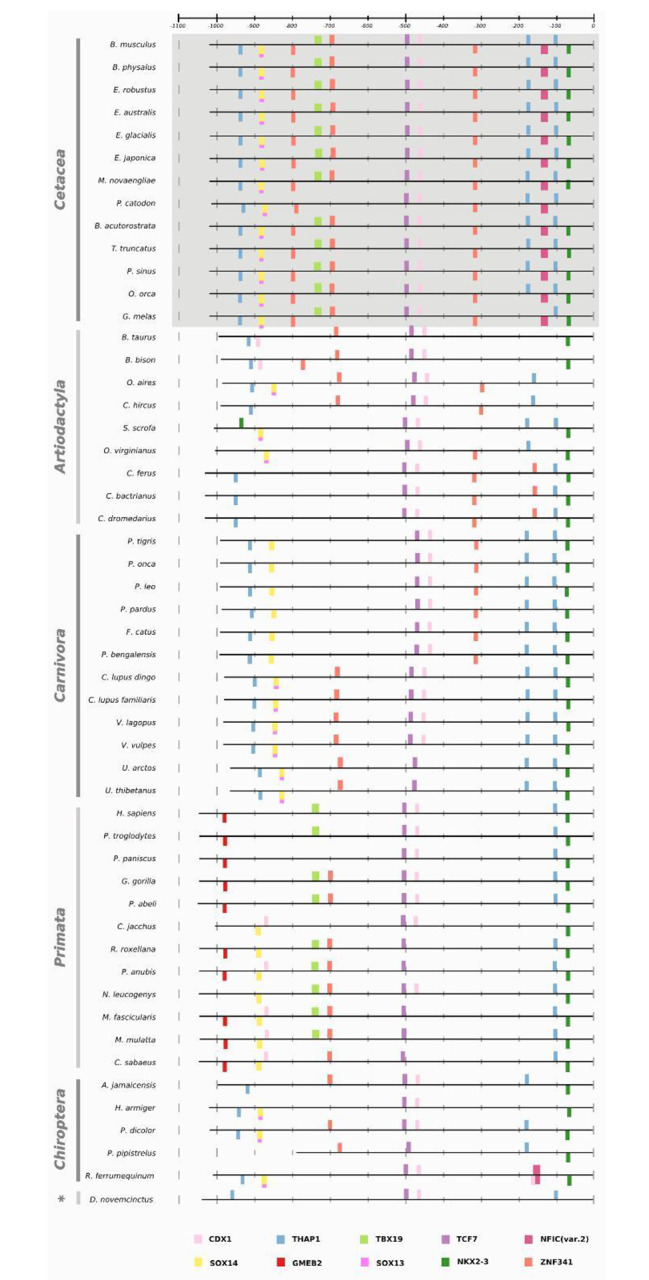
Enrichment pattern for the *EGF* promoter implemented in the Ciiider program. The result shows transcription factors in bars, such as TCF7 and CDX1, conserved in the phylogeny. Mammals are cetaceans, artiodactyls, carnivores, primates, bats, and cingulate (marked with an asterisk). The giant cetaceans are: *Balaenopteramusculus*, *Balaenoptera physalus*, *Eschrichtius robustus*, *Eubalaenaaustralis*, *Eubalaenaglacialis*, *Eubalaena japonica*, *Megaptera novaeangliae*, and *Physeter catodon*

**Fig. 4:**
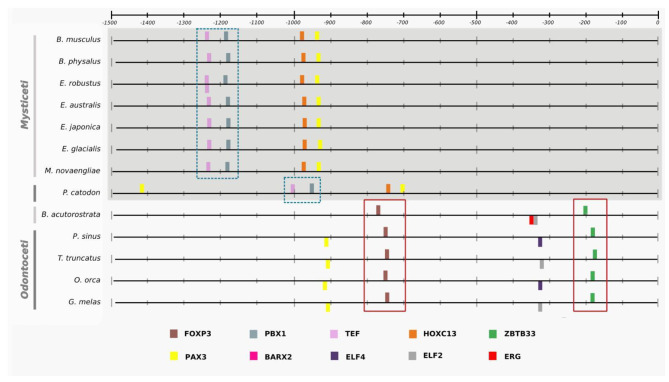
Closer look at the enrichment pattern for the *NCAPG* promoter implemented in the Ciiider program. The result shows transcription factors in bars. TEF and PBX1, highlighted in blue, are present only in *Physeter catodon*, *Eschrichtius robustus*, *Eubalaena japonica*, *Eubalaena australis*, *Eubalaena glacialis*, *Megaptera novaeangliae*, *Balaenoptera physalus*, and *Balaenoptera musculus* that are giant cetaceans, and FOXP3 and ZBTB33, highlighted in red, only in non-giant cetaceans such *Tursiops truncatus*, *Orcinus orca*, *Lipotes vexillifer*, *Phocoena sinus*, and *Balaenoptera acutorostrata scammoni*

**Fig. 5:**
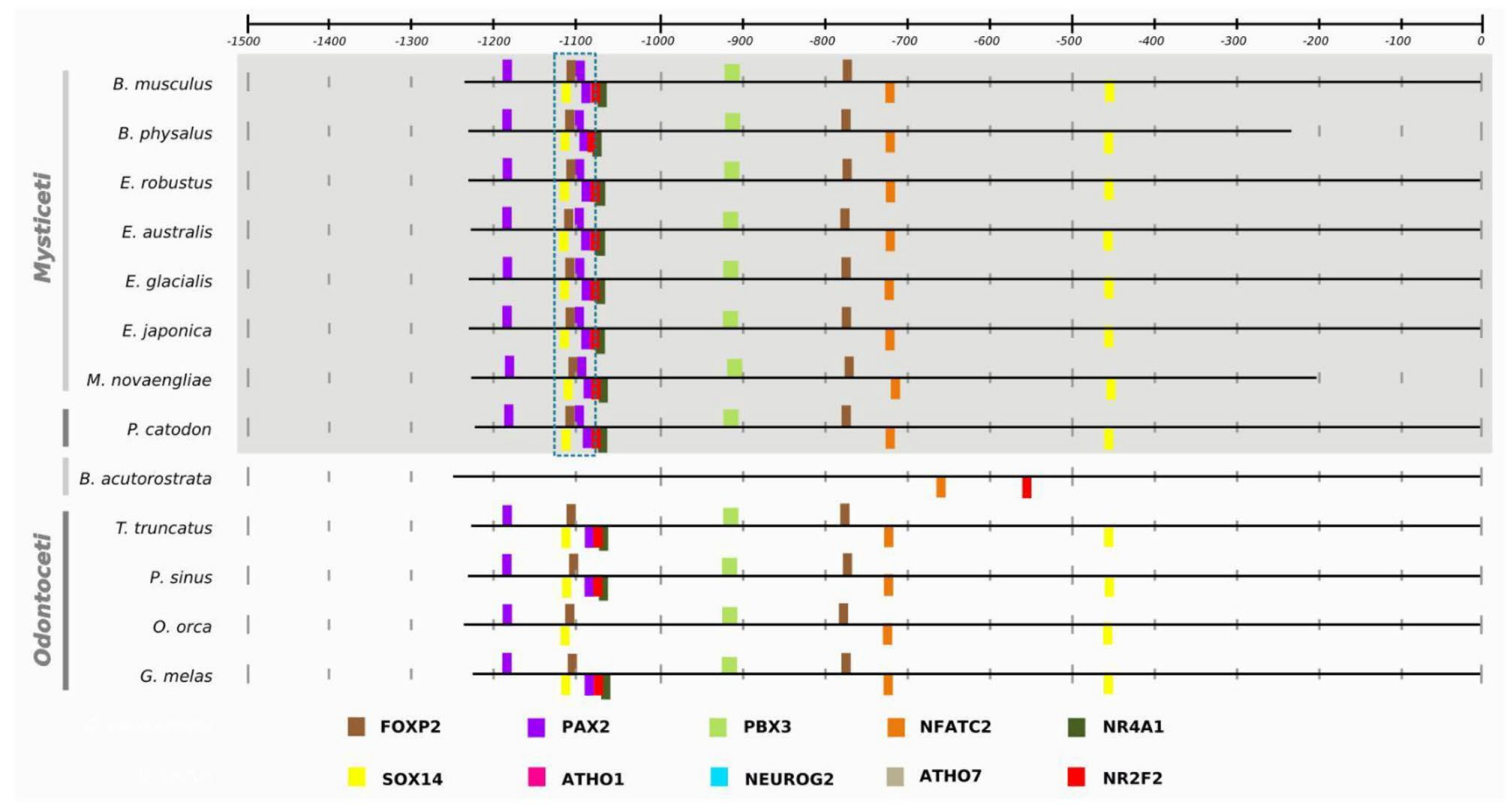
Closer look at the enrichment pattern for the *IGFBP7* promoter implemented in the Ciiider program. The result shows transcription factors in bars. PAX2, highlighted in blue, is present in a triple pattern only in *Physeter catodon*, *Eschrichtius robustus*, *Eubalaena japonica*, *Eubalaena australis*, *Eubalaena glacialis*, *Megaptera novaeangliae*, *Balaenoptera physalus*, and *Balaenoptera musculus*, which are classified as giants

To gain insights into the molecular evolution of non-coding regulatory regions of genes associated with body size in cetaceans, we employed a scanning approach using Ciiider to identify transcription factor binding sites (TFBS) for the nine promoters of interest across all species in our dataset. The identification of TFBS is crucial to understanding the regulatory mechanisms that control gene expression, and that may contribute to the evolution of morphological traits such as body size. By analyzing the presence and distribution of TFBS in these promoters, we aimed to identify potential regulatory modifications that may have contributed to gigantism in cetaceans and to gain a better understanding of the molecular basis of body size evolution in this group of mammals. The scanning approach performed in Ciider identified TFBS for all nine promoters in all species. The results of the enrichment analyses showed conservation patterns across different mammalian groups, suggesting that some transcription factors are evolutionarily conserved, as all mammals have the same patterns of transcription factor enrichment in the same approximate location of the promoter (Table 1, also Additional file 1: Supplementary Figures S9-S14 shows *GHSR*, *IGF2*, *IGFBP2*, *LCORL*, *PLAG1*, and *ZFAT*). For example, in the EGF promoter, TCF7 and CDX1 were found to be spatially conserved across phylogeny at the − 500 bp position within the promoter region, as shown in Fig. 3, which includes all species analysed to highlight this conservation. On the other hand, TFBS exclusive to certain groups was observed in the NCAPG and IGFBP7 promoters, as demonstrated in Figs. 4 and 5, respectively. Figures 4 and 5 only show cetaceans to highlight the specificity of the TFBS in the promoters related to giant and non-giant cetaceans. The complete figures, including other species, can be found in the supplementary material.

**Table 1:** The top ten over-represented transcription factors for each promoter of interest. These results are obtained when comparing the studied promoters against a background of human genes

Gene	Top ten over-represented transcription factors	
*EGF*	CDX1, GMEB2, NFIC, TCF7, SOX13, SOX14, NKZ2-3, TBX19, THAP1, ZNF341	
*GHSR*	CEBPB, FOXP2, KLF13, NR1D1, NR2F1, NR2F2, NR5A2, PPARG-RXRA, THRB, ZBTB7C	
*IGF2*	EGR3, PBX1, SP3, SP9, MLX, ZBTB14, ZNF282, NR2F1, PAX2, TFAP2A	
*IGFBP2*	BATFJUN, FOSL1, KLF17, SOX13, ZNF282, MYF6, NEUROG2, ZBTB14, NR2F2, ZNF341	
*IGFBP7*	ATOH7, ATOH1, PBX3, FOXP2, NEUROG2, NFATC2, NR2F2, NR4A1, PAX2, SOX14	
*LCORL*	ATOH7, ATOH1, PBX3, FOXP2, NEUROG2, NFATC2, NR2F2, NR4A1, PAX2, SOX14	
*NCAPG*	HOXC13, ELF4, ELF2, ERG, BARX2, ZBTB33, PAX3, PBX1, FOXP3, TEF	
*PLAG1*	ELF2, EGR3, ELF4, GATA6, PAX9, SP3, NR4A1, PBX3, SP9, TFAP2A	
*ZFAT*	FOSL1, FOSBJUN, FOSJUNB, FOXP2, BACH1, NRIH4, GMEB2, JDP2, NR2F2, RORB	

In the *NCAPG* promoter, we identified a pattern of enrichment that split cetaceans into two groups: giants and non-giants. The giant mysticetes had an enrichment pattern with the transcription factors TEF and PBX1 in the region between − 1300 and − 1200 bp positions, shared only with the sperm whale (*Physeter catodon*), a species of odontocete that can exceed 20 m in length. In contrast, all cetaceans not classified as giants showed the enrichment of the transcription factor FOXP3 in the − 800 bp position and the transcription factor ZBTB33 in the − 200 bp position.

Similar patterns were also found in the *IGFBP7* gene, with giant mysticetes presenting a unique triple enrichment pattern at the − 1100 bp position with the transcription factor PAX2 shared only with the sperm whale.

Additionally, we used phyloP from the PHAST package to estimate the molecular evolution rate of the promoters and identify signals of evolutionary acceleration in specific branches. Specifically, we aimed to identify whether promoters of giant cetaceans underwent accelerated evolution compared to non-giants. To achieve this, phyloP calculated the conservation and acceleration scores in a partitioned tree through a set of named branches, the giant cetaceans, and all remaining species .Thus, the tests for conservation/acceleration occur in the set of named branches relative to the others. Positive scores indicate conservation and negative scores indicate acceleration. A substitution model, against which all subtrees were compared, was derived from the phyloFit program, from the same PHAST package. Our analysis revealed possible evidence of accelerated evolution in the promoters of gigantic cetaceans, as evidenced by negative scores in the IGF2, IGFBP2, IGFBP7, and ZFAT promoters(Table 2).

**Table 2:** Conservation or acceleration in promoter sequences of the nine genes studied in this work, estimated based on the likelihood ratio test of phyloP for the subtree comparing an alternative model (alt_subscale) with a free scale parameter (alt_scale) within the given REV substitution model (null_scale). Positive scores indicate evolutionary conservation, and negative scores denote evolutionary acceleration, as observed in the *IGF2*, *IGFBP2*, *IGFBP7*, and *ZFAT* promoters

Gene	null_scale	alt_scale	alt_subscale	Log-likelihood ratio	score
EGF	0.98697	0.98845	0.91995	0.06824	0.71181
GHSR	0.99969	1.00026	0.97634	0.01678	0.85465
**IGF2**	**1.00693**	**1.00660**	**1.01574**	**0.00836**	**-0.89709**
**IGFBP2**	**0.85126**	**0.84986**	**1.11514**	**0.16139**	**-0.56994**
**IGFBP7**	0.97794	**0.97747**	**1.03213**	**0.01757**	**-0.85131**
LCORL	0.99179	0.99239	0.97383	0.01599	0.85809
NCAPG	0.99783	0.99838	0.92715	0.11610	0.62989
PLAG1	1.00465	1.00523	0.97163	0.01838	0.84795
**ZFAT**	**0.99639**	**0.99606**	**1.01425**	**0.01237**	**-0.87503**

## Discussion

This study investigates the molecular evolution of regulatory regions of genes potentially linked to cetaceans’ gigantism, focusing on the promoter region. We found evidence of enrichment of transcription factors binding sites potentially related to large body size, with distinct patterns between giant and non-giant cetaceans in the *IGFBP7* and *NCAPG* promoters. We also found evidence of acceleration in the *IGF2*, *IGFBP2*, *IGFBP7*, and *ZFAT* promoters. We will focus our discussion on these 5 promoters, as the other four (EGF, GHSR, LCORL, and PLAG1) did not yield relevant results for our research question.

Despite being non-coding regions, which are often known to be difficult to align and contain many neutrally evolving sites and potentially a few constrained ones, we obtained high-quality alignments from our promoters, with ultimately recovered phylogenetic trees consistent with known relationships among species, except for the NCAPG. In this case, the phylogenetic signal was strong enough for the sperm whale (*Physeter catodon*) odontocete to be grouped with the other mysticetes, excluding the minke whale, grouped with the other odontocetes. In this way, two clades of cetaceans are redeemed: one that contains only those classified as giants and the other with non-giant cetaceans. The use of the Bayesian approach also resulted in the formation of the same clades divided by size. The phylogenetic incongruence between the highly reliable species tree and the promoter tree is a common phenomenon across the Tree of Life, as different regions can have different evolutionary histories [21] due to mechanisms such as incomplete lineage sorting (ILS), introgression, or convergent evolution [22–24]. The last one, convergent evolution, could fit the scenario of this work since we have species from two evolutionarily distinct groups (odontocetes and mysticetes) with similar gigantism-related mechanisms. Moreover, as discussed in the following paragraph, the enrichment analysis provides evidence that the convergent evolution of this region is a plausible explanation for this case. Regarding the other eight promoters, the recovery of trees consistent with the most accepted phylogenetic hypotheses for the groups included in the study gives us more confidence that we are indeed using a fundamental regulatory region of the genes in our dataset. Additionally, as discussed further, the identification of evolutionarily conserved TFBS across different mammalian groups in our study further supports the functional importance and conservation of these regulatory regions.

The analyses implemented in the Ciiider program identified the transcription factors binding sites in promoters. Subsequently, the enrichment test revealed some patterns in the promoters of our dataset. First, the same transcription factors were found in the same approximate position in different mammalian lineages, demonstrating evolutionary conservation, and this is the case for the *EGF* promoter (Fig. 3). Regulatory elements spatially conserved among different lineages suggest an important biological role, as observed between humans and mice for the Cd247, a gene with functional consequences in systemic autoimmunity [25], and in transcription factors related to growth and development in monocot and dicot lineages [26]. Second, *NCAPG* and *IGFBP7* promoters presented different patterns for giant and non-giant cetaceans. In the NCAPG promoter, this transcription factors distribution pattern is likely responsible for the phylogenetic signal in the promoter tree discussed before. The sperm whale (*Physeter catodon*) has the transcription factors TEF and PBX1 in the region between − 1300 and − 1200 bp position like other giant mysticetes. In contrast, the minke whale has more similarities with smaller odontocetes than its giant mysticetes relatives. Our results suggest that these regions have undergone different selective pressures and that some of the TFBS may have evolved more rapidly in certain lineages. These findings provide further evidence that the NCAPG promoter has experienced unique evolutionary processes that could contribute to the observed incongruence in the phylogenetic tree.

TEF (Thyrotroph embryonic factor) is a protein that belongs to the proline- and acidic amino acid-rich (PAR) bZIP family and is expressed initially in the embryonic anterior pituitary, whereas in adults, it is involved in controlling the cell cycle and the death of hematopoietic cells [27, 28]. These features make TEF a possible tumor suppressor, as demonstrated in bladder cancer (BC). The upregulation significantly retarded BC cell growth by inhibiting the G1/S transition via regulating AKT/FOXOs signaling [28]. In the same way, PBX1 (Pre-B-cell leukemia homeobox 1) is a member of the Three Amino acid Loop Extension (TALE)-class homeodomain family. It is responsible for diverse developmental processes, including skeleton patterning, hematopoiesis, pancreas, and urogenital systems organogenesis [29–33]. It is also involved in fetal growth in activity with decidual natural killer (dNK) cells, driving transcription of pleiotrophin and osteoglycin in dNK cells. On the other hand, the PBX1 inactivation in mouse dNK cells impairs fetal development by decreasing growth-promoting factors that result in fetal growth restriction [34].

Together, both TEF and PBX1 factors are related to general growth processes, such as the control of cell proliferation or directly linked to embryonic growth like PBX1, highlighting the biological meaning of their enrichment pattern only in giant cetaceans, mainly when this enrichment occurs in the promoter of a gene strongly associated with increased body size, such as *NCAPG*.

The *NCAPG* (Non-SMC Condensin I Complex Subunit G) gene was previously associated with increased body size and weight gain in horses, donkeys, pigs, humans, and chickens [35–41]. In bovine species, evolutionarily close to the cetaceans, *NCAPG* is associated with many essential features such as birth weight, wither height, feeding efficiency, and pubertal growth [42–44]. In previous work from our group—focusing on coding regions—evolutionary analyses showed that the *NCAPG* gene has evidence of positive selection in giant cetaceans [45]. Our promoter and coding regions results imply this gene’s essential role in cetacean gigantism.

The *IGFBP7* promoter also showed a specific triple pattern transcription factor only shared by giant cetaceans: the PAX2 (Paired Box Gene 2), which is critical during the embryonic development of systems such as the central nervous system (brain and spinal cord), kidney, eye, ear, and urogenital tract [46, 47]. PAX2 deficiency has been associated with various growth defects, such as kidney hypoplasia, optic coloboma, and vesicoureteral reflux [48]. Furthermore, PAX2 role in embryo development and oncogenesis suggests that it works as a regulatory factor in cell growth [49, 50]. This feature is similar to the *IGFBP7* gene, a member of the *IGFBP* superfamily responsible for the viability of insulin-like growth factors (IGFs) that are molecules involved in promoting cell growth and division [51]. This gene also acts as an oncosuppressor in prostate, breast, lung, and colorectal cancer due to its regulatory action related to cell proliferation, cell adhesion, cell senescence, and angiogenesis [52–54]. One of the main challenges of gigantism is the suppression of tumors due to a large number of cells. Therefore, mechanisms that manage to mitigate cancerous processes were crucial during the evolutionary history of the giants.

The cetaceans not classified as giants in this work comprise *Tursiops truncatus*, *Orcinus orca*, *Lipotes vexillifer*, *Phocoena sinus*, and *Balaenoptera acutorostrata scammoni.* In the enrichment analyses performed in Ciiider, only these cetacean species share the transcription factors FOXP3 and ZBTB33 in the NCAPG promoter. The first, FOXP3 (Forkhead box protein P3), is a transcription factor belonging to the forkhead box protein family and may act as a transcriptional activator or repressor [55]. It is also associated with the differentiation and function of regulatory T (Treg) cells, which are responsible for suppressing the activation of other leukocytes and thus contribute to immune homeostasis [56–58].

The ZBTB33 (Zinc finger and BTB domain-containing 33, also known as Kaiso), exhibits bimodal DNA recognition and acts as a transcriptional repressor and activator depending on the sequence context and cellular phenotype [59]. As a repressor, it recruits other repressors, forming further complexes and aiding in dampening the transcription of the target gene by blocking the binding of transcriptional activators [60]. One of the targets of the transcriptional repressor action of ZBTB33 is the Wnt signaling pathway, associated with critical physiological activities such as growth, differentiation, and migration during development [61]. Focusing on growth, Wnt signaling shapes growing tissues while inducing cells to proliferate, acting as growth factors, and directly affecting cellular organization by the cytoskeleton and mitotic spindle [62]. In summary, the presence of transcription factors that can act as repressors in the promoter of the NCAPG gene related to body growth only in small cetaceans may indicate how these animals did not develop giant sizes.

We found evidence of accelerated evolution in *IGF2*, *IGFBP2*, *IGFBP7*, and *ZFAT* promoters. The first three (*IGF2*, *IGFBP2*, and *IGFBP7*) are a group of genes that work together to promote growth. The insulin-like growth factors (IGFs), such as *IGF2*, are important in somatic growth and cell proliferation and responsible for fetal and post-natal growth [63]. This action is only completed by the modulation of insulin-like growth factor binding proteins (*IGFBPs*), a group that serves as transport proteins for insulin-like growth factors, regulating the bioavailability and function of *IGFs* [64]. For this direct growth-promoting action, the evidence of evolution acceleration on the promoters found by phyloP in the giant cetaceans follows the knowledge about these genes and reinforces their coordinated performance. Furthermore, the *IGFBP7* coding sequence was also associated with positive selection in investigating gigantism in cetaceans [45]. Likewise, the ZFAT gene has been associated with height in multiple human populations in horse body size and has been reported to have crucial roles in the maintenance and differentiation of the adipocytes, the number of T cells, and embryonic development [65–68]. Therefore, they are likely associated with growth due to controlling various aspects of body enlargement and acting as tumor suppressors. The remaining promoters (EGF, GHSR, LCORL, NCAPG, and PLAG1) exhibit conservation, as identified by CiiiDER, which found highly conserved patterns in most of the genes of interest, with *NCAPG* and *IGFBP7* showing conservation specifically in cetacean groups. Notably, IGFBP7, which also underwent evolutionary acceleration as detected by phyloP, may be associated with multiple gene functions, including body growth and tumor suppression.

Recent studies in other lineages have also highlighted the importance of regulatory regions in controlling body size. For instance, a deletion in the promoter region of *IGF2BP1* has been associated with larger body sizes in chickens [69], and variation in the *STAT3* promoter has been shown to contribute to larger body size traits in cattle [70]. Additionally, the control of growth hormone *IGF1* protein levels by long non-coding RNA has been implicated in the size of large dogs [71]. These findings, along with our own, underscore the critical role that regulatory regions play in determining size characteristics across diverse taxa. Further studies on the molecular evolution of these regions are needed, and future experimental testing will provide further insights into the regulatory mechanisms underlying body size variation.

Although with some limitations, such as the number of genes used, our study provides the first steps toward what other works can reach, especially those related to experimental validation. It is far from the definitive answer to a complex question. Still, this start could be useful in future research, indicating which genes are possibly related to gigantism in cetaceans and that this phenomenon must be understood in an integrated way.

## Conclusions

We investigated the promoter regions of genes possibly associated with increased body size in giant cetaceans. In summary, we found evolutionary conservation and evidence of differential transcription factors enrichment, with distinct patterns between giants and non-giants cetaceans for *IGFBP7* and the *NCAPG* promoters. In *NCAPG*, observing the presence of repressive transcription factors only in cetaceans of small body-size was also possible. Furthermore, evolutionarily acceleration was detected in the promoters of the *IGF2*, *IGFBP2*, *IGFBP7*, and *ZFAT* genes. In conclusion, our study provides evidence of the evolution of cetacean gigantism from a regulatory approach.

## Materials and methods

### Sample data

The promoters of nine genes were chosen because they have been described in the scientific literature as associated with changes in body size. The *EGF* (Epidermal Growth Factor), *GHSR* (Growth Hormone Secretagogue Receptor), *IGF2* (Insulin-Like Growth Factor 2), *IGFBP2* (Insulin-Like Growth Factor Binding Protein 2), and *IGFBP7* (Insulin-Like Growth Factor Binding Protein 7) are part of the growth hormone/insulin-like growth factor (GH-IGF) axis, which plays a central role in regulating growth in vertebrates [72, 73]. The *LCORL* (Ligand Dependent Nuclear Receptor Corepressor Like), *NCAPG* (Non-SMC Condensin I Complex Subunit G), *PLAG1* (Pleomorphic Adenoma Gene 1), and *ZFAT* (Zinc Finger And AT-Hook Domain Containing) are associated with the body enlargement of species such as cows, pigs, sheep, and goats, which are artiodactyls, evolutionarily close to cetaceans [74, 75]. The sequences of these promoters were retrieved in the Eukaryotic Promoter Database (EPD) from the Swiss Institute of Bioinformatics. Firstly, we located the transcription start site (TSS) for the human species and selected a 1500 bp region upstream of the TSS. Then, the promoter sequences of cetacean and other mammalian species were searched in public databases, such as Ensembl and GenBank (NCBI), using BLAST (Basic Local Alignment Search Tool), which compares nucleotide or protein sequences and calculates the statistical significance, finding similarity regions among sequences of interest.

For cetaceans, we used sequences from 13 species, five odontocetes (*Tursiops truncatus, Orcinus orca, Lipotes vexillifer, Physeter catodon*, and *Phocoena sinus*), and eight mysticetes (*Balaenoptera acutorostrata scammoni, Eschrichtius robustus, Megaptera novaeangliae, Balaenoptera physalus, Balaenoptera musculus, Eubalaena australis, Eubalaena glacialis*, and *Eubalaena japonica*). The sequences for *Eubalaena australis* and *Eubalaena glacialis* were retrieved from genomes available on the public platform *DNA Zoo*. All other cetacean sequences were retrieved from GenBank, and the Additional file 1: Supplementary Table 1 shows the accession numbers.

Following Lambert et al. 2010 [76], gigantism is attributed to species larger than 10 m. In our dataset, the following species fit this definition: blue whale (*Balaenoptera musculus*), sperm whale (*Physeter catodon*), gray whale (*Eschrichtius robustus*), humpback whale (*Megaptera novaeangliae*), fin whale (*Balaenoptera physalus*), South Atlantic right whale (*Eubalaena australis*), North Atlantic right whale (*Eubalaena glacialis*), and Pacific right whale (*Eubalaena japonica*).

In addition to cetaceans, we included 39 other species to represent the major mammalian groups, such as the order Artiodactyla (*Bos taurus, Capra hircus, Bison bison, Odocoileus virginianus, Ovis aries, Sus scrofa, Camelus dromedarius, Camelus ferus, Camelus bactrianus*), Carnivora (*Panthera leo, Panthera onca, Panthera tigris altaica, Panthera pardus, Felis catus, Prionailurus bengalensis, Canis lupus familiaris, Canis lupus dingo, Vulpes lagopus, Vulpes vulpes, Ursus arctos horribilis, Ursus thibetanus*), Primate (*Homo sapiens, Pan paniscus, Pan troglodytes, Gorilla gorilla gorilla, Pongo abelii, Callithrix jacchus, Rhinopithecus roxellana, Papio anubis, Nomascus leucogenys, Macaca fascicularis, Macaca mulatta* and *Chlorocebus sabaeus)*, Cingulata *(Dasypus novemcinctus*), and Chiroptera (*Artibeus jamaicensis, Hipposideros armiger, Phyllostomus discolor, Pipistrellus pipistrellus, Rhinolophus ferrumequinum*). Thus, there were the same 52 species in each promoter studied.

### Phylogenetic reconstructions

The sequences were aligned using the MUSCLE program [77] and visualized in AliView [78]. After this, phylogenetic trees were constructed for each promoter using the IQ-TREE program’s maximum likelihood strategy, 1,000 bootstrap replicates to estimate branch confidence, 1,000 maximum iterations, 1,000 number of bootstrap alignments, 0.5 perturbation strength, 100 IQ-TREE stopping rule, 0.99 minimum correlation coefficient, and “auto” in substitution model. This entire process was done directly on the IQ-TREE Web Server portal [79]. For Bayesian analysis, we determine the optimal number of partitions and evolutionary models for each promoter using PARTITION FINDER software v2.1.1 [80], which employed the Bayesian Information Criterion (BIC). Subsequently, Bayesian phylogenetic trees were constructed using MrBayes v3.2.6 [81]. The Markov chain Monte Carlo (MCMC) algorithm was run for 5,000,000 generations with four chains, and trees were sampled every 100 generations, utilizing the molecular evolution model selected by PARTITION FINDER v2.1.1. The resulting trees were visualized using FigTree v1.3.1. Finally, we visualized the results in the program FigTree v1.3.1.

### Regulatory regions analyses

Promoter analyses were performed using Ciiider and phyloP tools. Ciiider was used to predict and to analyze transcription factor binding sites within a sequence and identify significantly enriched ones [82]. This is important since over-represented transcription factors are more likely to regulate gene expression that ultimately alters the phenotype [83]. We used scanning and enrichment approaches in Ciiider.

Given a sequence, the scanning predicts potential transcription factors in the region of interest. The MATCH algorithm searches for transcription factor binding sites in DNA sequences [84] using a Position Frequency Matrix (PFM). A set of non-redundant profiles derived from experimentally defined transcription factor binding sites for eukaryotes is used in this work, derived from the JASPAR database containing position matrixes of these elements [85]. Since PFMs generally have a highly conserved core-binding region flanked by areas of higher variability, a core PFM is created for the five most conserved consecutive bases. To search for transcription factor binding sites, sequences are divided into regions of five overlapping bases compared to the core PFM. If the similarity score between a five-base sequence and the core PFM meets a defined threshold, then the sequence window is increased to the full length of the transcription factors, and the similarity score to the full PFM is calculated. The default deficit is 0.15, meaning the scan will accept any transcription factors with MATCH scores of 0.85 or above [84].

The enrichment approach allow us to identify those transcription factor binding sites that are significantly over- or under-represented in the regions of interest when compared to the background regions used in the analysis. To reduce the possibility of chance findings, we used a comparative background consisting of several other genes provided by the Ciiider program. Thus, we reduce the chances of the results being stochastic. In short, Ciiider scans these background sequences using the same criteria for the sequences of interest and for the background sequences. The program determines the over- and under-representation of transcription factors by comparing the number of sequences containing these factors to those without them, followed by a statistical test such as Fisher’s exact test [84].

We used phyloP tool from PHAST (Phylogenetic Analysis with Space/Time) package to estimate the molecular evolution rate of the promoter and detect signals of evolutionary acceleration in specific branches [86, 87]. First, we generated a substitution model using the phyloFit program, which fits one or more tree models to multiple alignments of DNA sequences using maximum likelihood, and the substitution model used was REV (Reversible Evolutionary Model), the default of phyloFit, which is more realistic and flexible than simpler neutral models and can capture variations in nucleotide substitution rates at different positions in the alignment [88, 89]. Using REVl, we calculated conservation and acceleration scores with the “branch” option, which partitions the tree into named branches and tests for conservation/acceleration in the named branches relative to the others. We compared the set of named branches containing giant cetaceans against the remaining species. We selected the LRT option, which compares an alternative model having a free scale parameter with the substitution model, and the CONACC mode, which allows for acceleration as well as conservation, assigning positive values (scores) to indicate conservation and negative values to indicate acceleration. Thus, CONACC mode summarizes conservation and acceleration.

### Electronic supplementary material

Below is the link to the electronic supplementary material.

Supplementary Material 1: Additional file 1: Table S1: Species used in this study and the respective accession numbers from NCBI; Figure S1: Maximum likelihood tree generated by IQ − TREE constructed from the promoter region of the *EGF* gene. Numbers under nodes represent bootstrap support; Figure S2: Maximum likelihood tree generated by IQ − TREE constructed from the promoter region of the *GHSR* gene. Numbers under nodes represent bootstrap support; Figure S3: Maximum likelihood tree generated by IQ − TREE constructed from the promoter region of the *IGF2* gene. Numbers under nodes represent bootstrap support; Figure S4: Maximum likelihood tree generated by IQ − TREE constructed from the promoter region of the *IGFBP2* gene. Numbers under nodes represent bootstrap support; Figure S5: Maximum likelihood tree generated by IQ − TREE constructed from the promoter region of the *IGFBP7* gene. Numbers under nodes represent bootstrap support; Figure S6: Maximum likelihood tree generated by IQ − TREE constructed from the promoter region of the *LCORL* gene. Numbers under nodes represent bootstrap support; Figure S7: Maximum likelihood tree generated by IQ − TREE constructed from the promoter region of the *PLAG1* gene. Numbers under nodes represent bootstrap support; Figure S8: Maximum likelihood tree generated by IQ − TREE constructed from the promoter region of the *ZFAT* gene. Numbers under nodes represent bootstrap support; Figure S9: Enrichment pattern for the *GHSR* promoter implemented in the Ciiider program. The result shows transcription factors in bars. Mammals are cetaceans, artiodactyls, carnivores, primates, bats, and cingulates; Figure S10: Enrichment pattern for the *IGF2* promoter implemented in the Ciiider program. The result shows transcription factors in bars. Mammals are cetaceans, artiodactyls, carnivores, primates, bats, and cingulates; Figure S11: Enrichment pattern for the *IGFBP2* promoter implemented in the Ciiider program. The result shows transcription factors in bars. Mammals are cetaceans, artiodactyls, carnivores, primates, bats, and cingulates; Figure S12: Enrichment pattern for the *LCORL* promoter implemented in the Ciiider program. The result shows transcription factors in bars. Mammals are cetaceans, artiodactyls, carnivores, primates, bats, and cingulates; Figure S13: Enrichment pattern for the *PLAG1* promoter implemented in the Ciiider program. The result shows transcription factors in bars. Mammals are cetaceans, artiodactyls, carnivores, primates, bats, and cingulates; Figure S14: Enrichment pattern for the *ZFAT* promoter implemented in the Ciiider program. The result shows transcription factors in bars. Mammals are cetaceans, artiodactyls, carnivores, primates, bats, and cingulates.

## Data Availability

All the data supporting our findings are contained within the manuscript and in the supplemental file.

## Abbreviations

ACAN: Aggrecan
BLAST: Basic Local Alignment Search Tool
*CBS*: Cystathionine β – synthase
*EGF*: Epidermal Growth Factor
*EIF2AK3*: *T*ranslation initiation factor2-α kinase 3
EPD: Eukaryotic Promoter Database
*GHSR*: Growth Hormone Secretagogue Receptor
*GRB10*: Growth factor receptor-bound protein 10
*IGF*: Insulin-Like Growth Factor 2
*IGFBP2*: Insulin-Like Growth Factor Binding Protein 2
*IGFBP7*: Insulin-Like Growth Factor Binding Protein 7
*LCORL*: Ligand Dependent Nuclear Receptor Corepressor Like
*NCAPG*: Non-SMC Condensin I Complex Subunit G
*OBSL1*: *Obscurin-like protein 1*
PFM: Position Frequency Matrix
*PLAG1*: Pleomorphic Adenoma Gene 1
PLOD1: lysyl hydroxylase 1 gene
TSS: Transcription Start Site
*ZFAT*: Zinc Finger And AT-Hook Domain Containing

## References

[1] Mead JG, Brownell R. L. Order Cetacea in Mammal Species of the World: A Taxonomic and Geographic Reference (eds. Wilson, D. E. & Reeder, D. M.). United States of America: University Press, Cambridge; 2005;723–743.

[2] Thewissen J (2007). Whales originated from aquatic artiodactyls in the Eocene epoch of India. Nature.

[3] Berta A (2005). Marine mammals: evolutionary biology. United States of America.

[4] Leffler EM (2012). Revisiting an old riddle: what determines genetic diversity levels within species?. PLoS Biol.

[5] Damuth J (1981). Population density and body size in mammals. Nature.

[6] Jones ML, Swartz L. Gray Whale: Eschrichtius robustus. Encyclopedia of Marine Mammals 2nd edition. United States of America: Academic Press, Cambridge; 2009;503–511.

[7] Sears R, Perrin WF (2009). BlueWhale:Balaenopteramusculus. EncyclopediaofMarineMammals2ndedition.

[8] Downhower JF, Bulmer LS (1988). Calculating just how small a whale can be. Nature.

[9] Smith FA, Lyons SK (2011). How big should a mammal be? A macroecological look at mammalian body size over space and time. Phil Trans R Soc B.

[10] Goldbogen JA, Madsen PT (2018). The evolution of foraging capacity and gigantism in cetaceans. Exp Biol.

[11] Whitehead H. Sperm Whale: Physeter macrocephalus. Encyclopedia of Marine Mammals 3th edition. United States of America: Academic Press, Cambridge; 2018;919–925.

[12] Goldbogen JA (2019). Why whales are big but not bigger: physiological drivers and ecological limits in the age of ocean giants. Science.

[13] Sun Y (2019). Insights into body size variation in cetaceans from the evolution of body-size related genes. BMC Evol Bio.

[14] Carroll SB (2008). Evo-Devo and an expanding evolutionary synthesis: a genetic theory of morphological evolution. Cell.

[15] Dermitzakis ET, Clark AG (2002). Evolution of transcription factor binding sites in mammalian gene regulatory regions: conservation and turnover. Mol Biol Evol.

[16] Lowe CB (2011). Three periods of regulatory innovation during vertebrate evolution. Science.

[17] Karlsson EK (2007). Efficient mapping of mendelian traits in dogs through genome-wide association. Nat Genet.

[18] McGregor AP (2007). Morphological evolution through multiple cis-regulatory mutations at a single gene. Nature.

[19] Chan YF (2010). Adaptive evolution of pelvic reduction in sticklebacks by recurrent deletion of a Pitx1 enhancer. Science.

[20] McGowen MR (2020). Phylogenomic resolution of the Cetacean Tree of Life using Target sequence capture. Syst Biol.

[21] Degnan JH, Rosenberg NA (2006). Discordance of Species Trees with their most likely gene trees. PLoS Genet.

[22] Maddison WP (1997). Gene trees in species trees. Syst Biol.

[23] Satta Y (2000). DNA archives and our nearest relative: the trichotomy problem revisited. Mol Phylogenet Evol.

[24] Parker J (2013). Genome-wide signatures of convergent evolution in echolocating mammals. Nature.

[25] Pundhir S (2014). Spatially conserved regulatory elements identified within human and mouse Cd247 gene using high-throughput sequencing data from the ENCODE project. Gene.

[26] Xu F (2012). Cis-regulatory signatures of orthologous stress-associated bZIP transcription factors from rice, sorghum and Arabidopsis based on phylogenetic footprints. BMC Genom.

[27] Inukai T (2005). TEF, an antiapoptotic bZIP transcription factor related to the oncogenic E2A-HLF chimera, inhibits cell growth by down-regulating expression of the common β chain of cytokine receptors. Blood.

[28] Yang J (2019). Thyrotroph embryonic factor is downregulated in Bladder cancer and suppresses proliferation and tumorigenesis via the AKT/FOXOs signalling pathway. Cell Prolif.

[29] Selleri L (2001). Requirement for Pbx1 in skeletal patterning and programming chondrocyte proliferation and differentiation. Development.

[30] Specchia G (2001). Extramedullary involvement at relapse in acute promyelocytic Leukemia patients treated or not with all-trans retinoic acid: a report by the Gruppo Italiano Malattie Ematologiche dell’Adulto. J Clin Oncol.

[31] Kim SK (2002). Pbx1 inactivation disrupts pancreas development and in Ipf1-deficient mice promotes Diabetes Mellitus. Nat Genet.

[32] Schnabel CA (2003). Pbx1 is essential for adrenal development and urogenital differentiation. Genesis.

[33] Magnani L (2011). PBX1 genomic pioneer function drives ERα Signaling underlying progression in Breast Cancer. PLoS Genet.

[34] Zhou Y (2020). PBX1 expression in uterine natural killer cells drives fetal growth. Sci Transl Med.

[35] Tetens J (2013). A genome-wide association study indicates LCORL/NCAPG as a candidate locus for withers height in German Warmblood horses. Anim Genet.

[36] Shen J (2021). Genomic Analyses Reveal Distinct Genetic Architectures and selective pressures in Chinese donkeys. J Genet Genom.

[37] Rubin CJ, et al. Strong signatures of selection in the domestic pig genome. Proc. Natl. Acad. Sci. USA 2012;109:19529–19536. 10.1073/pnas.1217149109 PMC3511700 23151514

[38] Gudbjartsson DF (2008). Many sequence variants affecting diversity of adult human heigh. Nat Genet.

[39] Lettre G (2008). Identification of ten loci associated with height highlights new biological pathways in human growth. Nat Genet.

[40] Weedon MN (2008). Genome-wide association analysis identifies 20 loci that influence adult height. Nat Genet.

[41] Sasaki S (2004). Genetic mapping of quantitative trait loci affecting body weight, egg character and egg production in F2intercross chickens. Anim Genet.

[42] Eberlein A (2009). Dissection of genetic factors modulating fetal growth in cattle indicates a substantial role of the Non-SMC condensin I Complex, Subunit G (NCAPG) Gene. Genetics.

[43] Weikard R (2010). Metabolomic profiles indicate distinct physiological pathways affected by two loci with major divergent effect on Bos taurus growth and lipid deposition. Physiol Genom.

[44] Setoguchi K (2011). The SNP c.1326T > G in the non-SMC condensin I complex, subunit G (NCAPG) gene encoding a p.Ile442Met variant is associated with an increase in body frame size at puberty in cattle. Anim Genet.

[45] Silva FA (2022). The molecular evolution of genes previously associated with large sizes reveals possible pathways to cetacean gigantism. Sci Rep.

[46] Eccles MR (2002). PAX genes in development and Disease: the role of PAX2 in urogenital tract development. Int J Dev Biol.

[47] Porteous S (2000). Primary renal hypoplasia in humans and mice with PAX2 mutations: evidence of increased apoptosis in fetal kidneys of Pax21Neu +/– mutant mice. Hum Mol Genet.

[48] Weber S (2008). SIX2 and BMP4 mutations associate with anomalous kidney development. J Am Soc Nephrol.

[49] Jahangiri R (2018). PAX2 expression is correlated with better survival in tamoxifen-treated breast carcinoma patients. Tissue Cell.

[50] Song H (2013). PAX2 expression in Ovarian Cancer. Int J Mol Sci.

[51] Burger AM (2005). Essential roles of IGFBP-3 and IGFBP-rP1 in Breast cancer. Eur J Cancer.

[52] Akaogi K, et al. Specific accumulation of tumor-derived adhesion factor in tumor blood vessels and in capillary tube-like structures of cultured vascular endothelial cells. Proc. Natl. Acad. Sci. USA. 1996;93:8384–8389. 10.1073/pnas.93.16.8384 PMC38680 8710880

[53] Sprenger CC (1999). Insulin-like growth factor binding protein-related protein 1 (IGFBP-rP1) is a potential Tumor suppressor protein for Prostate cancer. Cancer Res.

[54] Wilson HM (2002). Insulin-like growth factor binding protein-related protein 1 inhibits proliferation of MCF-7 Breast cancer cells via a senescence-like mechanism. Cell Growth Differ.

[55] Jia H (2019). The expression of FOXP3 and its role in human cancers. Biochim Biophys Acta Rev Cancer.

[56] Hori S (2003). Control of regulatory T cell development by the transcription factor Foxp3. Science.

[57] Fontenot JD (2003). Foxp3 programs the development and function of CD4 + CD25 + regulatory T cells. Nat Immunol.

[58] Lu L (2017). The regulation of immune tolerance by FOXP3. Nature.

[59] Pozner A (2016). Cell specific Kaiso (ZBTB33) regulation of cell cycle through cyclin D1 and cyclin E1. J Biol Chem.

[60] Yoon HG (2003). N-CoR mediates DNA methylation-dependent repression through a methyl CpG binding protein Kaiso. Mol Cell.

[61] Azbazdar Y 1. Regulation of wnt signaling pathways at the plasma membrane and their Misregulation in Cancer. Front Cell Dev Biol. 2021. 10.3389/fcell.2021.631623. 10.3389/fcell.2021.631623 PMC7873896 33585487

[62] Nusse R, Clevers H (2017). Wnt/β-Catenin signaling, Disease, and emerging therapeutic modalities. Cell.

[63] Monzavi R, Cohen P (2002). IGFs and IGFBPs: role in health and Disease. Best Pract Res Clin Endocrinol Metab.

[64] Ding H, Wu T (2018). Insulin-like growth factor binding proteins in Autoimmune Diseases. Front Endocrinol.

[65] Takeuchi F (2009). Evaluation of genetic loci influencing adult height in the Japanese population. J Hum Genet.

[66] Allen HL (2010). Hundreds of variants clustered in genomic loci and biological pathways affect human height. Nature.

[67] Signer-Hasler H (2012). A genome-wide association study reveals loci influencing height and other conformation traits in horses. PLoS ONE.

[68] Makvandi-Nejad S (2012). Four loci explain 83% of size variation in the horse. PLoS ONE.

[69] Wang K (2021). The Chicken Pan-genome reveals Gene Content Variation and a promoter region deletion in IGF2BP1 affecting body size. Mol Biol Evol.

[70] Wu S (2018). Genetic variants in STAT3 promoter regions and their application in molecular breeding for body size traits in Qinchuan Cattle. Int J Mol Sci.

[71] Plassais J (2022). Natural and human-driven selection of a single non-coding body size variant in ancient and modern canids. Curr Biol.

[72] Canosa LF (2007). Neuroendocrine control of growth hormone in fish. Gen Comp Endocrinol.

[73] Bergan-Roller HE, Sheridan MA (2018). The growth hormone signaling system: insights into coordinating the anabolic and catabolic actions of growth hormone. Gen Comp Endocrinol.

[74] Takasuga A (2016). PLAG1 and NCAPG-LCORL in livestock. Anim Sci J.

[75] Wang W (2019). Molecular characterization and expression of SPP1, LAP3 and LCORL and their association with growth traits in sheep. Genes.

[76] Lambert O (2010). The giant bite of a new raptorial sperm whale from the Miocene epoch of Peru. Nature.

[77] Edgar RC, Muscle (2004). Multiple sequence alignment with high accuracy and high-throughput. Nucleic Acids Res.

[78] Larsson A, Aliview (2014). A fast and lightweight alignment viewer and editor for large data sets. Bioinfo.

[79] Nguyen LT (2015). IQ-TREE: a fast and effective stochastic algorithm for estimating maximum-likelihood phylogenies. Mol Biol Evol.

[80] Lanfear R (2016). PartitionFinder 2: new methods for selecting partitioned models of evolution for molecular and morphological phylogenetic analyses. Mol Biol Evol.

[81] Ronquist F, Huelsenbeck JP (2003). MrBayes 3: bayesian phylogenetic inference under mixed models. J Bioinform.

[82] Gearing LJ (2019). CiiiDER: a tool for predicting and analysing transcription factor binding sites. PLoS ONE.

[83] Boeva V (2016). Analysis of genomic sequence motifs for deciphering transcription factor binding and transcriptional regulation in eukaryotic cells. Front Genet.

[84] Kel AE (2003). MATCH: a tool for searching transcription factor binding sites in DNA sequences. Nucleic Acids Res.

[85] Castro-Mondragon, JA, et al. JASPAR 2020: Update of the open-access database of transcription factor binding profiles. Nucleic Acids Res. 2020;48:87–92. 10.1093/nar/gkz1001 PMC7145627 31701148

[86] Pollard KS (2010). Detection of nonneutral substitution rates on mammalian phylogenies. Genome Res.

[87] Hubisz MJ (2011). PHAST and RPHAST: phylogenetic analysis with space/time models. Brief Bioinform.

[88] Siepel A, Pollard KS, Haussler D (2006). New methods for detecting lineage-specific selection. Comput Mol Biol.

[89] Siepel A, Haussler D (2004). Phylogenetic estimation of context-dependent substitution rates by maximum likelihood. Mol Biol Evol.

