# Supplementary material for: Patterns of enrichment and acceleration in evolutionary rates of promoters suggest a role of regulatory regions in cetacean gigantism
Source: BMC Ecol Evol. 2023 Oct 24;23:62. doi: 10.1186/s12862-023-02171-5 (PMC10594719; doi:10.1186/s12862-023-02171-5)
Supplement: Supplementary file 1 — Supplementary Material 1: Additional file 1: Table S1: Species used in this study and the respective accession numbers from NCBI; Figure S1: Maximum likelihood tree generated by IQ − TREE constructed from the promoter region of the EGF gene. Numbers under nodes represent bootstrap support; Figure S2: Maximum likelihood tree generated by IQ − TREE constructed from the promoter region of the GHSR gene. Numbers under nodes represent bootstrap support; Figure S3: Maximum likelihood tree generated by IQ − TREE constructed from the promoter region of the IGF2 gene. Numbers under nodes represent bootstrap support; Figure S4: Maximum likelihood tree generated by IQ − TREE constructed from the promoter region of the IGFBP2 gene. Numbers under nodes represent bootstrap support; Figure S5: Maximum likelihood tree generated by IQ − TREE constructed from the promoter region of the IGFBP7 gene. Numbers under nodes represent bootstrap support; Figure S6: Maximum likelihood tree generated by IQ − TREE constructed from the promoter region of the LCORL gene. Numbers under nodes represent bootstrap support; Figure S7: Maximum likelihood tree generated by IQ − TREE constructed from the promoter region of the PLAG1 gene. Numbers under nodes represent bootstrap support; Figure S8: Maximum likelihood tree generated by IQ − TREE constructed from the promoter region of the ZFAT gene. Numbers under nodes represent bootstrap support; Figure S9: Enrichment pattern for the GHSR promoter implemented in the Ciiider program. The result shows transcription factors in bars. Mammals are cetaceans, artiodactyls, carnivores, primates, bats, and cingulates; Figure S10: Enrichment pattern for the IGF2 promoter implemented in the Ciiider program. The result shows transcription factors in bars. Mammals are cetaceans, artiodactyls, carnivores, primates, bats, and cingulates; Figure S11: Enrichment pattern for the IGFBP2 promoter implemented in the Ciiider program. The result shows transcription factors in b [file 12862_2023_2171_MOESM1_ESM.docx]

**SUPPLEMENTARY MATERIAL**

**Patterns of enrichment and acceleration in evolutionary rates of promoters suggest a role of regulatory regions in cetacean gigantism**

Silva Felipe André¹, Picorelli Agnello César Rios¹, Veiga Giovanna Selleghin¹, Nery Mariana Freitas¹*

¹ Dept of Genetics, Evolution, Microbiology & Immunology, Institute of Biology, University of Campinas, Rua Monteiro Lobato, 255 Campinas/SP 13083-862

* marinery@unicamp.br; Tel.: + 55 21 3938-7356

**Table S1**. Species used in this study and the respective accession numbers from NCBI.

| Gene | Order | Species | Common name | NCBI accession number |
| --- | --- | --- | --- | --- |
| **EGF** | Chiroptera | *Artibeus jamaicensis* | Jamaican fruit-eating bat | XM_037156199.1 |
|  | Cetartiodactyla | *Balaenoptera acutorostrata* | Minke whale | XM_007191032.1 |
|  | Cetartiodactyla | *Balaenoptera musculus* | Blue whale | XM_036852782.1 |
|  | Cetartiodactyla | *Balaenoptera physalus* | Fin whale | GCA_008795845.1 |
|  | Cetartiodactyla | *Bison bison* | Plains bison | XM_010858536.1 |
|  | Cetartiodactyla | *Bos taurus* | Cow | XM_024993778.1 |
|  | Primates | *Callithrix jacchus* | White-tufted-ear marmoset | XM_002745488.5 |
|  | Cetartiodactyla | *Camelus bactrianus* | Bactrian Camel | XM_010953680.1 |
|  | Cetartiodactyla | *Camelus dromedarius* | Arabian Camel | XM_010981085.2 |
|  | Cetartiodactyla | *Camelus ferus* | Wild Bactrian camel | XM_032461807.1 |
|  | Carnivora | *Canis lupus dingo* | Dingo | XM_025465917.3 |
|  | Carnivora | *Canis lupus familiaris* | Dog | NM_001003094.3 |
|  | Cetartiodactyla | *Capra hircus* | Goat | XM_018049910.1 |
|  | Primates | *Chlorocebus sabaeus* | Green Monkey | XM_007999539.2 |
|  | Cingulata | *Dasypus novemcinctus* | Nine-banded armadillo | XM_004476167.1 |
|  | Certartiodactyla | *Eschrichtius robustus* | Gray whale | GCA_004363415.1 |
|  | Cetartiodactyla | *Eubalaena japonica* | North Pacific right whale | GCA_004363455.1 |
|  | Carnivora | *Felis catus* | Domestic cat | XM_006930906.3 |
|  | Cetartiodactyla | *Globicephala melas* | Long-finned pilot whale | XM_030864324.1 |
|  | Primates | *Gorilla gorilla* | Western Gorilla | XM_019026087.2 |
|  | Chiroptera | *Hipposideros armiger* | Great roundleaf bat | XM_019635993.1 |
|  | Primates | *Homo sapiens* | Human | NM_001178130.3 |
|  | Primates | *Macaca fascicularis* | Crab-eating macaque | XM_005555679.2 |
|  | Primates | *Macaca mulatta* | Rhesus monkey | XM_002804174.3 |
|  | Cetartiodactyla | *Megaptera novaeangliae* | Humpback whale | GCA_004329385.1 |
|  | Primates | *Nomascus leucogenys* | Northern white-cheeked gibbon | XM_030798013.1 |
|  | Cetartiodactyla | *Odocoileus virginianus texanus* | White-tailed deer | XM_020887922.1 |
|  | Cetartiodactyla | *Orcinus orca* | Killer whale | XM_004269572.2 |
|  | Cetartiodactyla | *Ovis aries* | Sheep | GCA_022416915.1 |
|  | Primates | *Pan paniscus* | Pygmy chimpanzee | XM_008955391.2 |
|  | Primates | *Pan troglodytes* | Chimpanzee | XM_016952025.2 |
|  | Carnivora | *Panthera leo* | Lion | XM_042935684.1 |
|  | Carnivora | *Panthera onca* | Jaguar | GCA_004023805.1 |
|  | Carnivora | *Panthera pardus* | Leopard | XM_019417575.1 |
|  | Carnivora | *Panthera tigris* | Tiger | XM_042984035.1 |
|  | Primates | *Papio anubis* | Olive baboon | XM_003899090.4 |
|  | Cetartiodactyla | *Phocoena sinus* | Vaquita | XM_032633399.1 |
|  | Chiroptera | *Phyllostomus discolor* | Pale spear-nosed bat | XM_036025296.1 |
|  | Cetartiodactyla | *Physeter catodon* | Sperm whale | XM_007105851.2 |
|  | Chiroptera | *Pipistrellus pipistrellus* | Common pipistrelle | GCA_903992545.1 |
|  | Primates | *Pongo abelii* | Sumatran orangutan | XM_002815059.3 |
|  | Carnivora | *Prionailurus bengalensis* | Leopard cat | XM_043570436.1 |
|  | Chiroptera | *Rhinolophus ferrumequinum* | Greater horseshoe bat | XM_033105271.1 |
|  | Primates | *Rhinopithecus roxellana* | Golden snub-nosed monkey | XM_010389070.1 |
|  | Cetartiodactyla | *Sus scrofa* | Pig | NM_214020.2 |
|  | Cetartiodactyla | *Tursiops truncatus* | Common bottlenose dolphin | XM_019928058.2 |
|  | Carnivora | *Ursus arctos* | Brown bear | XM_026499184.3 |
|  | Carnivora | *Ursus thibetanus* | Japanese black bear | GCA_014364545.1 |
|  | Carnivora | *Vulpes lagopus* | Arctic fox | XM_041758096.1 |
|  | Carnivora | *Vulpes vulpes* | Red fox | XM_026017613.1 |
| **GHSR** | Chiroptera | *Artibeus jamaicensis* | Jamaican fruit-eating bat | XM_037137498.1 |
|  | Cetartiodactyla | *Balaenoptera acutorostrata* | Minke whale | XM_007197500.1 |
|  | Cetartiodactyla | *Balaenoptera musculus* | Blue whale | XM_036850806.1 |
|  | Cetartiodactyla | *Balaenoptera physalus* | Fin whale | GCA_008795845.1 |
|  | Cetartiodactyla | *Bison bison* | Plains bison | XM_010835263.1 |
|  | Cetartiodactyla | *Bos taurus* | Cow | NM_001143736.2 |
|  | Primates | *Callithrix jacchus* | White-tufted-ear marmoset | NM_001257223.1 |
|  | Cetartiodactyla | *Camelus bactrianus* | Bactrian Camel | XM_010947779.1 |
|  | Cetartiodactyla | *Camelus dromedarius* | Arabian Camel | XM_031454849.1 |
|  | Cetartiodactyla | *Camelus ferus* | Wild Bactrian camel | XM_006188097.1 |
|  | Carnivora | *Canis lupus dingo* | Dingo | XM_025425603.3 |
|  | Carnivora | *Canis lupus familiaris* | Dog | NM_001099945.1 |
|  | Cetartiodactyla | *Capra hircus* | Goat | XM_018047679.1 |
|  | Primates | *Chlorocebus sabaeus* | Green Monkey | XM_037988562.1 |
|  | Cingulata | *Dasypus novemcinctus* | Nine-banded armadillo | XM_004463952.1 |
|  | Certartiodactyla | *Eschrichtius robustus* | Gray whale | GCA_004363415.1 |
|  | Cetartiodactyla | *Eubalaena japonica* | North Pacific right whale | GCA_004363455.1 |
|  | Carnivora | *Felis catus* | Domestic cat | XM_023260870.1 |
|  | Cetartiodactyla | *Globicephala melas* | Long-finned pilot whale | XM_030863506.1 |
|  | Primates | *Gorilla gorilla* | Western Gorilla | XM_004038010.3 |
|  | Chiroptera | *Hipposideros armiger* | Great roundleaf bat | XM_019624324.1 |
|  | Primates | *Homo sapiens* | Human | NM_198407.2 |
|  | Primates | *Macaca fascicularis* | Crab-eating macaque | XM_005546393.3 |
|  | Primates | *Macaca mulatta* | Rhesus monkey | XM_001084886.4 |
|  | Cetartiodactyla | *Megaptera novaeangliae* | Humpback whale | GCA_004329385.1 |
|  | Primates | *Nomascus leucogenys* | Northern white-cheeked gibbon | XM_003256467.2 |
|  | Cetartiodactyla | *Odocoileus virginianus texanus* | White-tailed deer | XM_020879608.1 |
|  | Cetartiodactyla | *Orcinus orca* | Killer whale | XM_004270543.3 |
|  | Cetartiodactyla | *Ovis aries* | Sheep | NM_001009760.1 |
|  | Primates | *Pan paniscus* | Pygmy chimpanzee | XM_003831941.2 |
|  | Primates | *Pan troglodytes* | Chimpanzee | XM_003310101.4 |
|  | Carnivora | *Panthera leo* | Lion | XM_042903326.1 |
|  | Carnivora | *Panthera onca* | Jaguar | GCA_004023805.1 |
|  | Carnivora | *Panthera pardus* | Leopard | XM_019463185.1 |
|  | Carnivora | *Panthera tigris* | Tiger | XM_007077104.3 |
|  | Primates | *Papio anubis* | Olive baboon | XM_021934664.2 |
|  | Cetartiodactyla | *Phocoena sinus* | Vaquita | XM_032630793.1 |
|  | Chiroptera | *Phyllostomus discolor* | Pale spear-nosed bat | XM_036017704.1 |
|  | Cetartiodactyla | *Physeter catodon* | Sperm whale | XM_007122099.1 |
|  | Chiroptera | *Pipistrellus pipistrellus* | Common pipistrelle | GCA_903992545.1 |
|  | Primates | *Pongo abelii* | Sumatran orangutan | XM_002814288.3 |
|  | Carnivora | *Prionailurus bengalensis* | Leopard cat | XM_043596067.1 |
|  | Chiroptera | *Rhinolophus ferrumequinum* | Greater horseshoe bat | XM_033089514.1 |
|  | Primates | *Rhinopithecus roxellana* | Golden snub-nosed monkey | XM_010356909.2 |
|  | Cetartiodactyla | *Sus scrofa* | Pig | NM_214180.1 |
|  | Cetartiodactyla | *Tursiops truncatus* | Common bottlenose dolphin | XM_004328327.3 |
|  | Carnivora | *Ursus arctos* | Brown bear | XM_026500544.1 |
|  | Carnivora | *Ursus thibetanus* | Japanese black bear | GCA_014364545.1 |
|  | Carnivora | *Vulpes lagopus* | Arctic fox | XM_041731504.1 |
|  | Carnivora | *Vulpes vulpes* | Red fox | XM_026007762.1 |
| **IGF2** | Chiroptera | *Artibeus jamaicensis* | Jamaican fruit-eating bat | XM_037156355.1 |
|  | Cetartiodactyla | *Balaenoptera acutorostrata* | Minke whale | XM_007171159.2 |
|  | Cetartiodactyla | *Balaenoptera musculus* | Blue whale | XM_036860921.1 |
|  | Cetartiodactyla | *Balaenoptera physalus* | Fin whale | GCA_023338255.1 |
|  | Cetartiodactyla | *Bison bison* | Plains bison | XM_010838191.1 |
|  | Cetartiodactyla | *Bos taurus* | Cow | XM_005227270.4 |
|  | Primates | *Callithrix jacchus* | White-tufted-ear marmoset | XM_017978384.2 |
|  | Cetartiodactyla | *Camelus bactrianus* | Bactrian Camel | XM_045517984.1 |
|  | Cetartiodactyla | *Camelus dromedarius* | Arabian Camel | XM_031447933.1 |
|  | Cetartiodactyla | *Camelus ferus* | Wild Bactrian camel | XM_032490153.1 |
|  | Carnivora | *Canis lupus dingo* | Dingo | XM_035701918.1 |
|  | Carnivora | *Canis lupus familiaris* | Dog | XM_038423442.1 |
|  | Cetartiodactyla | *Capra hircus* | Goat | NM_001287042.1 |
|  | Primates | *Chlorocebus sabaeus* | Green Monkey | XM_037998825.1 |
|  | Cingulata | *Dasypus novemcinctus* | Nine-banded armadillo | XM_023585640.1 |
|  | Certartiodactyla | *Eschrichtius robustus* | Gray whale | GCA_004363415.1 |
|  | Cetartiodactyla | *Eubalaena japonica* | North Pacific right whale | GCA_004363455.1 |
|  | Carnivora | *Felis catus* | Domestic cat | XM_023240192.1 |
|  | Cetartiodactyla | *Globicephala melas* | Long-finned pilot whale | XM_030833134.1 |
|  | Primates | *Gorilla gorilla* | Western Gorilla | XM_019036290.2 |
|  | Chiroptera | *Hipposideros armiger* | Great roundleaf bat | XM_019667379.1 |
|  | Primates | *Homo sapiens* | Human | NM_000612.6 |
|  | Primates | *Macaca fascicularis* | Crab-eating macaque | XM_005595745.2 |
|  | Primates | *Macaca mulatta* | Rhesus monkey | XM_028833047.1 |
|  | Cetartiodactyla | *Megaptera novaeangliae* | Humpback whale | GCA_004329385.1 |
|  | Primates | *Nomascus leucogenys* | Northern white-cheeked gibbon | XM_030811478.1 |
|  | Cetartiodactyla | *Odocoileus virginianus texanus* | White-tailed deer | XM_020883289.1 |
|  | Cetartiodactyla | *Orcinus orca* | Killer whale | XM_033402551.1 |
|  | Cetartiodactyla | *Ovis aries* | Sheep | NM_001009311.1 |
|  | Primates | *Pan paniscus* | Pygmy chimpanzee | XM_024930725.2 |
|  | Primates | *Pan troglodytes* | Chimpanzee | XM_024346903.1 |
|  | Carnivora | *Panthera leo* | Lion | XM_042906212.1 |
|  | Carnivora | *Panthera onca* | Jaguar | GCA_004023805.1 |
|  | Carnivora | *Panthera pardus* | Leopard | XM_019437038.1 |
|  | Carnivora | *Panthera tigris* | Tiger | XM_042958947.1 |
|  | Primates | *Papio anubis* | Olive baboon | NM_001169030.1 |
|  | Cetartiodactyla | *Phocoena sinus* | Vaquita | XM_032640906.1 |
|  | Chiroptera | *Phyllostomus discolor* | Pale spear-nosed bat | XM_036029860.1 |
|  | Cetartiodactyla | *Physeter catodon* | Sperm whale | XM_024117562.2 |
|  | Chiroptera | *Pipistrellus pipistrellus* | Common pipistrelle | GCA_903992545.1 |
|  | Primates | *Pongo abelii* | Sumatran orangutan | XM_024254895.1 |
|  | Carnivora | *Prionailurus bengalensis* | Leopard cat | XM_043582018.1 |
|  | Chiroptera | *Rhinolophus ferrumequinum* | Greater horseshoe bat | XM_033119574.1 |
|  | Primates | *Rhinopithecus roxellana* | Golden snub-nosed monkey | XM_030917925.1 |
|  | Cetartiodactyla | *Sus scrofa* | Pig | XM_021080603.1 |
|  | Cetartiodactyla | *Tursiops truncatus* | Common bottlenose dolphin | XM_033861440.1 |
|  | Carnivora | *Ursus arctos* | Brown bear | XM_048216642.1 |
|  | Carnivora | *Ursus thibetanus* | Japanese black bear | GCA_014364545.1 |
|  | Carnivora | *Vulpes lagopus* | Arctic fox | XM_041722161.1 |
|  | Carnivora | *Vulpes vulpes* | Red fox | XM_026004183.1 |
| **IGFBP2** | Chiroptera | *Artibeus jamaicensis* | Jamaican fruit-eating bat | XM_037132277.1 |
|  | Cetartiodactyla | *Balaenoptera acutorostrata* | Minke whale | XM_007188015.2 |
|  | Cetartiodactyla | *Balaenoptera musculus* | Blue whale | XM_036858004.1 |
|  | Cetartiodactyla | *Balaenoptera physalus* | Fin whale | GCA_023338255.1 |
|  | Cetartiodactyla | *Bison bison* | Plains bison | XM_010861286.1 |
|  | Cetartiodactyla | *Bos taurus* | Cow | NM_174555.1 |
|  | Primates | *Callithrix jacchus* | White-tufted-ear marmoset | XM_035305635.1 |
|  | Cetartiodactyla | *Camelus bactrianus* | Bactrian Camel | XM_045512012.1 |
|  | Cetartiodactyla | *Camelus dromedarius* | Arabian Camel | XM_031452126.1 |
|  | Cetartiodactyla | *Camelus ferus* | Wild Bactrian camel | XM_032480190.1 |
|  | Carnivora | *Canis lupus dingo* | Dingo | XM_025441712.3 |
|  | Carnivora | *Canis lupus familiaris* | Dog | XM_038447898.1 |
|  | Cetartiodactyla | *Capra hircus* | Goat | NM_001314299.1 |
|  | Primates | *Chlorocebus sabaeus* | Green Monkey | XM_007966218.2 |
|  | Cingulata | *Dasypus novemcinctus* | Nine-banded armadillo | XM_004469597.3 |
|  | Certartiodactyla | *Eschrichtius robustus* | Gray whale | GCA_004363415.1 |
|  | Cetartiodactyla | *Eubalaena japonica* | North Pacific right whale | GCA_004363455.1 |
|  | Carnivora | *Felis catus* | Domestic cat | XM_011285583.3 |
|  | Cetartiodactyla | *Globicephala melas* | Long-finned pilot whale | XM_030852597.1 |
|  | Primates | *Gorilla gorilla* | Western Gorilla | XM_019022087.1 |
|  | Chiroptera | *Hipposideros armiger* | Great roundleaf bat | XM_019638357.1 |
|  | Primates | *Homo sapiens* | Human | NM_000597.3 |
|  | Primates | *Macaca fascicularis* | Crab-eating macaque | XM_005574235.3 |
|  | Primates | *Macaca mulatta* | Rhesus monkey | XM_015110935.2 |
|  | Cetartiodactyla | *Megaptera novaeangliae* | Humpback whale | GCA_004329385.1 |
|  | Primates | *Nomascus leucogenys* | Northern white-cheeked gibbon | XM_003254057.3 |
|  | Cetartiodactyla | *Odocoileus virginianus texanus* | White-tailed deer | XM_020913292.1 |
|  | Cetartiodactyla | *Orcinus orca* | Killer whale | XM_004262739.2 |
|  | Cetartiodactyla | *Ovis aries* | Sheep | NM_001009436.1 |
|  | Primates | *Pan paniscus* | Pygmy chimpanzee | XM_003818692.3 |
|  | Primates | *Pan troglodytes* | Chimpanzee | XM_516234.6 |
|  | Carnivora | *Panthera leo* | Lion | XM_042950107.1 |
|  | Carnivora | *Panthera onca* | Jaguar | GCA_004023805.1 |
|  | Carnivora | *Panthera pardus* | Leopard | XM_019446040.1 |
|  | Carnivora | *Panthera tigris* | Tiger | XM_042995299.1 |
|  | Primates | *Papio anubis* | Olive baboon | XM_017946941.3 |
|  | Cetartiodactyla | *Phocoena sinus* | Vaquita | XM_032637023.1 |
|  | Chiroptera | *Phyllostomus discolor* | Pale spear-nosed bat | XM_028510641.2 |
|  | Cetartiodactyla | *Physeter catodon* | Sperm whale | XM_028497083.1 |
|  | Chiroptera | *Pipistrellus pipistrellus* | Common pipistrelle | GCA_903992545.1 |
|  | Primates | *Pongo abelii* | Sumatran orangutan | XM_003780700.3 |
|  | Carnivora | *Prionailurus bengalensis* | Leopard cat | XM_043577772.1 |
|  | Chiroptera | *Rhinolophus ferrumequinum* | Greater horseshoe bat | XM_033112685.1 |
|  | Primates | *Rhinopithecus roxellana* | Golden snub-nosed monkey | XM_010362314.2 |
|  | Cetartiodactyla | *Sus scrofa* | Pig | NM_214003.1 |
|  | Cetartiodactyla | *Tursiops truncatus* | Common bottlenose dolphin | XM_033859797.1 |
|  | Carnivora | *Ursus arctos* | Brown bear | XM_026491989.2 |
|  | Carnivora | *Ursus thibetanus* | Japanese black bear | GCA_014364545.1 |
|  | Carnivora | *Vulpes lagopus* | Arctic fox | XM_041737343.1 |
|  | Carnivora | *Vulpes vulpes* | Red fox | XM_026002193.1 |
| **IGFBP7** | Chiroptera | *Artibeus jamaicensis* | Jamaican fruit-eating bat | XM_037162274.1 |
|  | Cetartiodactyla | *Balaenoptera acutorostrata* | Minke whale | XM_007165296.1 |
|  | Cetartiodactyla | *Balaenoptera musculus* | Blue whale | GCA_009873245.3 |
|  | Cetartiodactyla | *Balaenoptera physalus* | Fin whale | GCA_023338255.1 |
|  | Cetartiodactyla | *Bison bison* | Plains bison | XM_010859718.1 |
|  | Cetartiodactyla | *Bos taurus* | Cow | NM_001102300.2 |
|  | Primates | *Callithrix jacchus* | White-tufted-ear marmoset | XM_002745806.5 |
|  | Cetartiodactyla | *Camelus bactrianus* | Bactrian Camel | XM_010967517.1 |
|  | Cetartiodactyla | *Camelus dromedarius* | Arabian Camel | XM_010983378.2 |
|  | Cetartiodactyla | *Camelus ferus* | Wild Bactrian camel | XM_032459617.1 |
|  | Carnivora | *Canis lupus dingo* | Dingo | XM_025435809.2 |
|  | Carnivora | *Canis lupus familiaris* | Dog | XM_038685739.1 |
|  | Cetartiodactyla | *Capra hircus* | Goat | XM_018049499.1 |
|  | Primates | *Chlorocebus sabaeus* | Green Monkey | XM_037989407.1 |
|  | Cingulata | *Dasypus novemcinctus* | Nine-banded armadillo | XM_023586179.1 |
|  | Certartiodactyla | *Eschrichtius robustus* | Gray whale | GCA_004363415.1 |
|  | Cetartiodactyla | *Eubalaena japonica* | North Pacific right whale | GCA_004363455.1 |
|  | Carnivora | *Felis catus* | Domestic cat | XM_003985355.5 |
|  | Cetartiodactyla | *Globicephala melas* | Long-finned pilot whale | XM_030880663.1 |
|  | Primates | *Gorilla gorilla* | Western Gorilla | XM_019025243.2 |
|  | Chiroptera | *Hipposideros armiger* | Great roundleaf bat | XM_019664367.1 |
|  | Primates | *Homo sapiens* | Human | NM_001553.3 |
|  | Primates | *Macaca fascicularis* | Crab-eating macaque | XM_005555209.3 |
|  | Primates | *Macaca mulatta* | Rhesus monkey | XM_001083041.4 |
|  | Cetartiodactyla | *Megaptera novaeangliae* | Humpback whale | GCA_004329385.1 |
|  | Primates | *Nomascus leucogenys* | Northern white-cheeked gibbon | XM_030818887.1 |
|  | Cetartiodactyla | *Odocoileus virginianus texanus* | White-tailed deer | XM_020873868.1 |
|  | Cetartiodactyla | *Orcinus orca* | Killer whale | XM_004268245.2 |
|  | Cetartiodactyla | *Ovis aries* | Sheep | NM_001145181.1 |
|  | Primates | *Pan paniscus* | Pygmy chimpanzee | XM_003806499.4 |
|  | Primates | *Pan troglodytes* | Chimpanzee | XM_517274.7 |
|  | Carnivora | *Panthera leo* | Lion | XM_042936292.1 |
|  | Carnivora | *Panthera onca* | Jaguar | GCA_004023805.1 |
|  | Carnivora | *Panthera pardus* | Leopard | XM_019469010.1 |
|  | Carnivora | *Panthera tigris* | Tiger | XM_007075885.2 |
|  | Primates | *Papio anubis* | Olive baboon | XM_003898849.4 |
|  | Cetartiodactyla | *Phocoena sinus* | Vaquita | XM_032633730.1 |
|  | Chiroptera | *Phyllostomus discolor* | Pale spear-nosed bat | XM_028507809.2 |
|  | Cetartiodactyla | *Physeter catodon* | Sperm whale | XM_024132019.2 |
|  | Chiroptera | *Pipistrellus pipistrellus* | Common pipistrelle | GCA_903992545.1 |
|  | Primates | *Pongo abelii* | Sumatran orangutan | XM_002814758.4 |
|  | Carnivora | *Prionailurus bengalensis* | Leopard cat | XM_043604216.1 |
|  | Chiroptera | *Rhinolophus ferrumequinum* | Greater horseshoe bat | XM_033105544.1 |
|  | Primates | *Rhinopithecus roxellana* | Golden snub-nosed monkey | XM_010389440.2 |
|  | Cetartiodactyla | *Sus scrofa* | Pig | NM_001163801.1 |
|  | Cetartiodactyla | *Tursiops truncatus* | Common bottlenose dolphin | XM_019928113.2 |
|  | Carnivora | *Ursus arctos* | Brown bear | XM_026508963.1 |
|  | Carnivora | *Ursus thibetanus* | Japanese black bear | GCA_014364545.1 |
|  | Carnivora | *Vulpes lagopus* | Arctic fox | XM_041725589.1 |
|  | Carnivora | *Vulpes vulpes* | Red fox | XM_026003499.1 |
| **LCORL** | Chiroptera | *Artibeus jamaicensis* | Jamaican fruit-eating bat | XM_037132755.1 |
|  | Cetartiodactyla | *Balaenoptera acutorostrata* | Minke whale | XM_007187617.1 |
|  | Cetartiodactyla | *Balaenoptera musculus* | Blue whale | GCA_009873245.3 |
|  | Cetartiodactyla | *Balaenoptera physalus* | Fin whale | GCA_023338255.1 |
|  | Cetartiodactyla | *Bison bison* | Plains bison | XM_010860204.1 |
|  | Cetartiodactyla | *Bos taurus* | Cow | XM_024993368.1 |
|  | Primates | *Callithrix jacchus* | White-tufted-ear marmoset | XM_035293964.1 |
|  | Cetartiodactyla | *Camelus bactrianus* | Bactrian Camel | XM_045508726.1 |
|  | Cetartiodactyla | *Camelus dromedarius* | Arabian Camel | XM_031436803.1 |
|  | Cetartiodactyla | *Camelus ferus* | Wild Bactrian camel | XM_032494561.1 |
|  | Carnivora | *Canis lupus dingo* | Dingo | XM_049108034.1 |
|  | Carnivora | *Canis lupus familiaris* | Dog | XM_038662104.1 |
|  | Cetartiodactyla | *Capra hircus* | Goat | XM_018049322.1 |
|  | Primates | *Chlorocebus sabaeus* | Green Monkey | XM_008017795.2 |
|  | Cingulata | *Dasypus novemcinctus* | Nine-banded armadillo | XM_004474219.3 |
|  | Certartiodactyla | *Eschrichtius robustus* | Gray whale | GCA_004363415.1 |
|  | Cetartiodactyla | *Eubalaena japonica* | North Pacific right whale | GCA_004363455.1 |
|  | Carnivora | *Felis catus* | Domestic cat | XM_023253272.2 |
|  | Cetartiodactyla | *Globicephala melas* | Long-finned pilot whale | XM_030864288.1 |
|  | Primates | *Gorilla gorilla* | Western Gorilla | XM_031009938.1 |
|  | Chiroptera | *Hipposideros armiger* | Great roundleaf bat | XM_019654226.1 |
|  | Primates | *Homo sapiens* | Human | NM_001166139.2 |
|  | Primates | *Macaca fascicularis* | Crab-eating macaque | XM_045392180.1 |
|  | Primates | *Macaca mulatta* | Rhesus monkey | XM_028848137.1 |
|  | Cetartiodactyla | *Megaptera novaeangliae* | Humpback whale | GCA_004329385.1 |
|  | Primates | *Nomascus leucogenys* | Northern white-cheeked gibbon | XM_030801148.1 |
|  | Cetartiodactyla | *Odocoileus virginianus texanus* | White-tailed deer | XM_020878311.1 |
|  | Cetartiodactyla | *Orcinus orca* | Killer whale | XM_033410644.1 |
|  | Cetartiodactyla | *Ovis aries* | Sheep | XM_042251264.1 |
|  | Primates | *Pan paniscus* | Pygmy chimpanzee | XM_034958347.1 |
|  | Primates | *Pan troglodytes* | Chimpanzee | XM_009447409.3 |
|  | Carnivora | *Panthera leo* | Lion | XM_042936809.1 |
|  | Carnivora | *Panthera onca* | Jaguar | GCA_004023805.1 |
|  | Carnivora | *Panthera pardus* | Leopard | XM_019453616.1 |
|  | Carnivora | *Panthera tigris* | Tiger | XM_042984861.1 |
|  | Primates | *Papio anubis* | Olive baboon | XM_017958610.3 |
|  | Cetartiodactyla | *Phocoena sinus* | Vaquita | XM_032631881.1 |
|  | Chiroptera | *Phyllostomus discolor* | Pale spear-nosed bat | XM_036019053.1 |
|  | Cetartiodactyla | *Physeter catodon* | Sperm whale | XM_028492102.1 |
|  | Chiroptera | *Pipistrellus pipistrellus* | Common pipistrelle | GCA_903992545.1 |
|  | Primates | *Pongo abelii* | Sumatran orangutan | XM_023206984.3 |
|  | Carnivora | *Prionailurus bengalensis* | Leopard cat | XM_043572875.1 |
|  | Chiroptera | *Rhinolophus ferrumequinum* | Greater horseshoe bat | XM_033105883.1 |
|  | Primates | *Rhinopithecus roxellana* | Golden snub-nosed monkey | XM_030922726.1 |
|  | Cetartiodactyla | *Sus scrofa* | Pig | XM_021100293.1 |
|  | Cetartiodactyla | *Tursiops truncatus* | Common bottlenose dolphin | XM_019928429.2 |
|  | Carnivora | *Ursus arctos* | Brown bear | XM_044389739.2 |
|  | Carnivora | *Ursus thibetanus* | Japanese black bear | GCA_014364545.1 |
|  | Carnivora | *Vulpes lagopus* | Arctic fox | XM_041751213.1 |
|  | Carnivora | *Vulpes vulpes* | Red fox | XM_025997171.1 |
| **NCAPG** | Chiroptera | *Artibeus jamaicensis* | Jamaican fruit-eating bat | XM_037132754.1 |
|  | Cetartiodactyla | *Balaenoptera acutorostrata* | Minke whale | XM_007187616.2 |
|  | Cetartiodactyla | *Balaenoptera musculus* | Blue whale | XM_036852212.1 |
|  | Cetartiodactyla | *Balaenoptera physalus* | Fin whale | GCA_008795845.1 |
|  | Cetartiodactyla | *Bison bison* | Plains bison | XM_010860211.1 |
|  | Cetartiodactyla | *Bos taurus* | Cow | NM_001102376.2 |
|  | Primates | *Callithrix jacchus* | White-tufted-ear marmoset | XM_002745953.5 |
|  | Cetartiodactyla | *Camelus bactrianus* | Bactrian Camel | XM_010973021.2 |
|  | Cetartiodactyla | *Camelus dromedarius* | Arabian Camel | XM_010991029.2 |
|  | Cetartiodactyla | *Camelus ferus* | Wild Bactrian camel | XM_014566119.2 |
|  | Carnivora | *Canis lupus dingo* | Dingo | XM_025437798.2 |
|  | Carnivora | *Canis lupus familiaris* | Dog | XM_038662116.1 |
|  | Cetartiodactyla | *Capra hircus* | Goat | XM_018049327.1 |
|  | Primates | *Chlorocebus sabaeus* | Green Monkey | XM_038008668.1 |
|  | Cingulata | *Dasypus novemcinctus* | Nine-banded armadillo | XM_004474216.2 |
|  | Certartiodactyla | *Eschrichtius robustus* | Gray whale | GCA_004363415.1 |
|  | Cetartiodactyla | *Eubalaena japonica* | North Pacific right whale | GCA_004363455.1 |
|  | Carnivora | *Felis catus* | Domestic cat | XM_003985501.6 |
|  | Cetartiodactyla | *Globicephala melas* | Long-finned pilot whale | XM_030864302.1 |
|  | Primates | *Gorilla gorilla* | Western Gorilla | XM_004038484.3 |
|  | Chiroptera | *Hipposideros armiger* | Great roundleaf bat | XM_019654234.1 |
|  | Primates | *Homo sapiens* | Human | NM_022346.5 |
|  | Primates | *Macaca fascicularis* | Crab-eating macaque | XM_005554547.3 |
|  | Primates | *Macaca mulatta* | Rhesus monkey | XM_015138088.2 |
|  | Cetartiodactyla | *Megaptera novaeangliae* | Humpback whale | GCA_004329385.1 |
|  | Primates | *Nomascus leucogenys* | Northern white-cheeked gibbon | XM_003258518.3 |
|  | Cetartiodactyla | *Odocoileus virginianus texanus* | White-tailed deer | XM_020878320.1 |
|  | Cetartiodactyla | *Orcinus orca* | Killer whale | XM_004282517.3 |
|  | Cetartiodactyla | *Ovis aries* | Sheep | XM_027970895.2 |
|  | Primates | *Pan paniscus* | Pygmy chimpanzee | XM_008952160.3 |
|  | Primates | *Pan troglodytes* | Chimpanzee | XM_016951398.1 |
|  | Carnivora | *Panthera leo* | Lion | XM_042936823.1 |
|  | Carnivora | *Panthera onca* | Jaguar | GCA_004023805.1 |
|  | Carnivora | *Panthera pardus* | Leopard | XM_019453623.1 |
|  | Carnivora | *Panthera tigris* | Tiger | XM_007093746.3 |
|  | Primates | *Papio anubis* | Olive baboon | XM_009206580.2 |
|  | Cetartiodactyla | *Phocoena sinus* | Vaquita | XM_032631894.1 |
|  | Chiroptera | *Phyllostomus discolor* | Pale spear-nosed bat | XM_028505733.2 |
|  | Cetartiodactyla | *Physeter catodon* | Sperm whale | XM_007101874.3 |
|  | Chiroptera | *Pipistrellus pipistrellus* | Common pipistrelle | GCA_903992545.1 |
|  | Primates | *Pongo abelii* | Sumatran orangutan | XM_009239834.2 |
|  | Carnivora | *Prionailurus bengalensis* | Leopard cat | XM_043572880.1 |
|  | Chiroptera | *Rhinolophus ferrumequinum* | Greater horseshoe bat | XM_033105897.1 |
|  | Primates | *Rhinopithecus roxellana* | Golden snub-nosed monkey | XM_010368009.2 |
|  | Cetartiodactyla | *Sus scrofa* | Pig | XM_005666548.3 |
|  | Cetartiodactyla | *Tursiops truncatus* | Common bottlenose dolphin | XM_019928433.2 |
|  | Carnivora | *Ursus arctos* | Brown bear | XM_026514505.1 |
|  | Carnivora | *Ursus thibetanus* | Japanese black bear | GCA_014364545.1 |
|  | Carnivora | *Vulpes lagopus* | Arctic fox | XM_041751214.1 |
|  | Carnivora | *Vulpes vulpes* | Red fox | XM_025997157.1 |
| **PLAG1** | Chiroptera | *Artibeus jamaicensis* | Jamaican fruit-eating bat | XM_037167719.1 |
|  | Cetartiodactyla | *Balaenoptera acutorostrata* | Minke whale | XM_007168591.1 |
|  | Cetartiodactyla | *Balaenoptera musculus* | Blue whale | GCA_009873245.3 |
|  | Cetartiodactyla | *Balaenoptera physalus* | Fin whale | GCA_008795845.1 |
|  | Cetartiodactyla | *Bison bison* | Plains bison | XM_010838971.1 |
|  | Cetartiodactyla | *Bos taurus* | Cow | XM_005215432.3 |
|  | Primates | *Callithrix jacchus* | White-tufted-ear marmoset | XM_002758917.4 |
|  | Cetartiodactyla | *Camelus bactrianus* | Bactrian Camel | XM_010970685.2 |
|  | Cetartiodactyla | *Camelus dromedarius* | Arabian Camel | XM_031441602.1 |
|  | Cetartiodactyla | *Camelus ferus* | Wild Bactrian camel | XM_006191052.3 |
|  | Carnivora | *Canis lupus dingo* | Dingo | XM_025477769.3 |
|  | Carnivora | *Canis lupus familiaris* | Dog | XM_038441197.1 |
|  | Cetartiodactyla | *Capra hircus* | Goat | XM_013969222.2 |
|  | Primates | *Chlorocebus sabaeus* | Green Monkey | XM_008000683.2 |
|  | Cingulata | *Dasypus novemcinctus* | Nine-banded armadillo | XM_023586203.1 |
|  | Certartiodactyla | *Eschrichtius robustus* | Gray whale | GCA_004363415.1 |
|  | Cetartiodactyla | *Eubalaena japonica* | North Pacific right whale | GCA_004363455.1 |
|  | Carnivora | *Felis catus* | Domestic cat | XM_006943223.5 |
|  | Cetartiodactyla | *Globicephala melas* | Long-finned pilot whale | XM_030859722.1 |
|  | Primates | *Gorilla gorilla* | Western Gorilla | XM_019031869.2 |
|  | Chiroptera | *Hipposideros armiger* | Great roundleaf bat | XM_019664519.1 |
|  | Primates | *Homo sapiens* | Human | NM_001114634.2 |
|  | Primates | *Macaca fascicularis* | Crab-eating macaque | XM_005563346.2 |
|  | Primates | *Macaca mulatta* | Rhesus monkey | XM_001085625.4 |
|  | Cetartiodactyla | *Megaptera novaeangliae* | Humpback whale | GCA_004329385.1 |
|  | Primates | *Nomascus leucogenys* | Northern white-cheeked gibbon | XM_003255987.2 |
|  | Cetartiodactyla | *Odocoileus virginianus texanus* | White-tailed deer | XM_020900828.1 |
|  | Cetartiodactyla | *Orcinus orca* | Killer whale | XM_033437622.1 |
|  | Cetartiodactyla | *Ovis aries* | Sheep | XM_012183720.3 |
|  | Primates | *Pan paniscus* | Pygmy chimpanzee | XM_003823234.2 |
|  | Primates | *Pan troglodytes* | Chimpanzee | XM_003311815.4 |
|  | Carnivora | *Panthera leo* | Lion | XM_042923689.1 |
|  | Carnivora | *Panthera onca* | Jaguar | GCA_004023805.1 |
|  | Carnivora | *Panthera pardus* | Leopard | XM_019421298.1 |
|  | Carnivora | *Panthera tigris* | Tiger | XM_042973166.1 |
|  | Primates | *Papio anubis* | Olive baboon | XM_003902772.5 |
|  | Cetartiodactyla | *Phocoena sinus* | Vaquita | XM_032609352.1 |
|  | Chiroptera | *Phyllostomus discolor* | Pale spear-nosed bat | XM_028533682.2 |
|  | Cetartiodactyla | *Physeter catodon* | Sperm whale | XM_024127070.2 |
|  | Chiroptera | *Pipistrellus pipistrellus* | Common pipistrelle | GCA_903992545.1 |
|  | Primates | *Pongo abelii* | Sumatran orangutan | XM_009243818.2 |
|  | Carnivora | *Prionailurus bengalensis* | Leopard cat | XM_043601105.1 |
|  | Chiroptera | *Rhinolophus ferrumequinum* | Greater horseshoe bat | XM_033126801.1 |
|  | Primates | *Rhinopithecus roxellana* | Golden snub-nosed monkey | XM_010362535.2 |
|  | Cetartiodactyla | *Sus scrofa* | Pig | XM_021089354.1 |
|  | Cetartiodactyla | *Tursiops truncatus* | Common bottlenose dolphin | XM_019925318.2 |
|  | Carnivora | *Ursus arctos* | Brown bear | XM_026516455.3 |
|  | Carnivora | *Ursus thibetanus* | Japanese black bear | GCA_014364545.1 |
|  | Carnivora | *Vulpes lagopus* | Arctic fox | XM_041769644.1 |
|  | Carnivora | *Vulpes vulpes* | Red fox | XM_026011675.1 |
| **ZFAT** | Chiroptera | *Artibeus jamaicensis* | Jamaican fruit-eating bat | XM_037166717.1 |
|  | Cetartiodactyla | *Balaenoptera acutorostrata* | Minke whale | XM_007188909.1 |
|  | Cetartiodactyla | *Balaenoptera musculus* | Blue whale | XM_036830778.1 |
|  | Cetartiodactyla | *Balaenoptera physalus* | Fin whale | GCA_023338255.1 |
|  | Cetartiodactyla | *Bison bison* | Plains bison | XM_010834786.1 |
|  | Cetartiodactyla | *Bos taurus* | Cow | XM_005215319.4 |
|  | Primates | *Callithrix jacchus* | White-tufted-ear marmoset | XM_035277373.1 |
|  | Cetartiodactyla | *Camelus bactrianus* | Bactrian Camel | XM_010972675.1 |
|  | Cetartiodactyla | *Camelus dromedarius* | Arabian Camel | XM_031439667.1 |
|  | Cetartiodactyla | *Camelus ferus* | Wild Bactrian camel | XM_032467923.1 |
|  | Carnivora | *Canis lupus dingo* | Dingo | XM_035701771.1 |
|  | Carnivora | *Canis lupus familiaris* | Dog | XM_038685079.1 |
|  | Cetartiodactyla | *Capra hircus* | Goat | XM_018058597.1 |
|  | Primates | *Chlorocebus sabaeus* | Green Monkey | XM_008001598.2 |
|  | Cingulata | *Dasypus novemcinctus* | Nine-banded armadillo | XM_012528645.2 |
|  | Certartiodactyla | *Eschrichtius robustus* | Gray whale | GCA_004363415.1 |
|  | Cetartiodactyla | *Eubalaena japonica* | North Pacific right whale | GCA_004363455.1 |
|  | Carnivora | *Felis catus* | Domestic cat | XM_019823173.3 |
|  | Cetartiodactyla | *Globicephala melas* | Long-finned pilot whale | XM_030875874.1 |
|  | Primates | *Gorilla gorilla* | Western Gorilla | XM_031014020.1 |
|  | Chiroptera | *Hipposideros armiger* | Great roundleaf bat | XM_019651281.1 |
|  | Primates | *Homo sapiens* | Human | NM_020863.4 |
|  | Primates | *Macaca fascicularis* | Crab-eating macaque | XM_005564124.3 |
|  | Primates | *Macaca mulatta* | Rhesus monkey | XM_015146034.2 |
|  | Cetartiodactyla | *Megaptera novaeangliae* | Humpback whale | GCA_004329385.1 |
|  | Primates | *Nomascus leucogenys* | Northern white-cheeked gibbon | XM_003276274.3 |
|  | Cetartiodactyla | *Odocoileus virginianus texanus* | White-tailed deer | XM_020893093.1 |
|  | Cetartiodactyla | *Orcinus orca* | Killer whale | XM_033419751.1 |
|  | Cetartiodactyla | *Ovis aries* | Sheep | XM_027972875.2 |
|  | Primates | *Pan paniscus* | Pygmy chimpanzee | XM_003830075.3 |
|  | Primates | *Pan troglodytes* | Chimpanzee | XM_016959895.2 |
|  | Carnivora | *Panthera leo* | Lion | XM_042923643.1 |
|  | Carnivora | *Panthera onca* | Jaguar | GCA_004023805.1 |
|  | Carnivora | *Panthera pardus* | Leopard | XM_019459783.1 |
|  | Carnivora | *Panthera tigris* | Tiger | XM_042973108.1 |
|  | Primates | *Papio anubis* | Olive baboon | XM_017962334.3 |
|  | Cetartiodactyla | *Phocoena sinus* | Vaquita | XM_032610134.1 |
|  | Chiroptera | *Phyllostomus discolor* | Pale spear-nosed bat | XM_028534076.2 |
|  | Cetartiodactyla | *Physeter catodon* | Sperm whale | XM_028500261.1 |
|  | Chiroptera | *Pipistrellus pipistrellus* | Common pipistrelle | GCA_903992545.1 |
|  | Primates | *Pongo abelii* | Sumatran orangutan | XM_024251400.1 |
|  | Carnivora | *Prionailurus bengalensis* | Leopard cat | XM_043601834.1 |
|  | Chiroptera | *Rhinolophus ferrumequinum* | Greater horseshoe bat | XM_033126444.1 |
|  | Primates | *Rhinopithecus roxellana* | Golden snub-nosed monkey | XM_010380280.2 |
|  | Cetartiodactyla | *Sus scrofa* | Pig | XM_021088872.1 |
|  | Cetartiodactyla | *Tursiops truncatus* | Common bottlenose dolphin | XM_033842738.1 |
|  | Carnivora | *Ursus arctos* | Brown bear | XM_026495374.3 |
|  | Carnivora | *Ursus thibetanus* | Japanese black bear | GCA_014364545.1 |
|  | Carnivora | *Vulpes lagopus* | Arctic fox | XM_041769041.1 |
|  | Carnivora | *Vulpes vulpes* | Red fox | XM_025993483.1 |


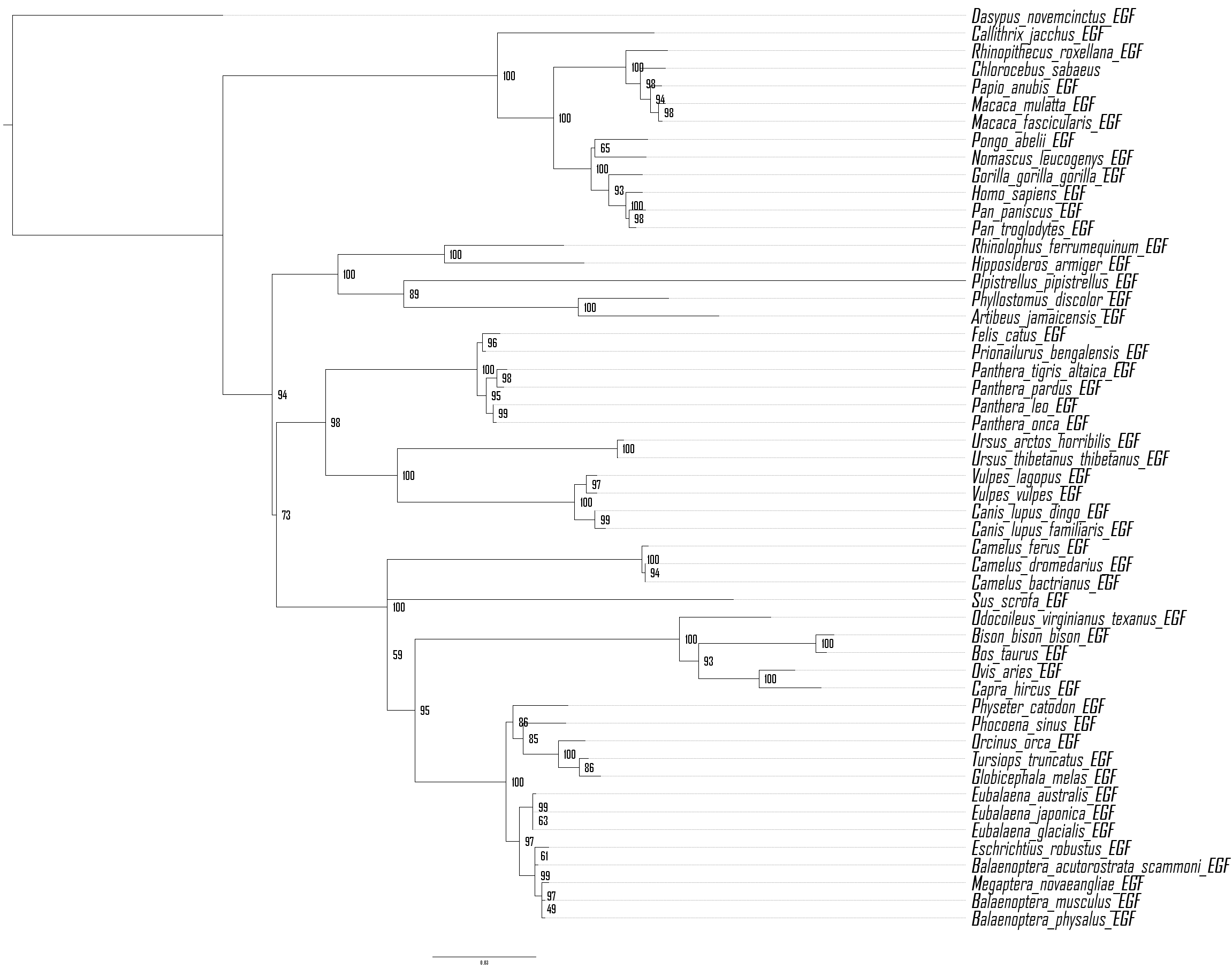


**Figure S1**: Maximum likelihood tree generated by IQ−TREE constructed from the promoter region of the EGF gene. Numbers under nodes represent bootstrap support.


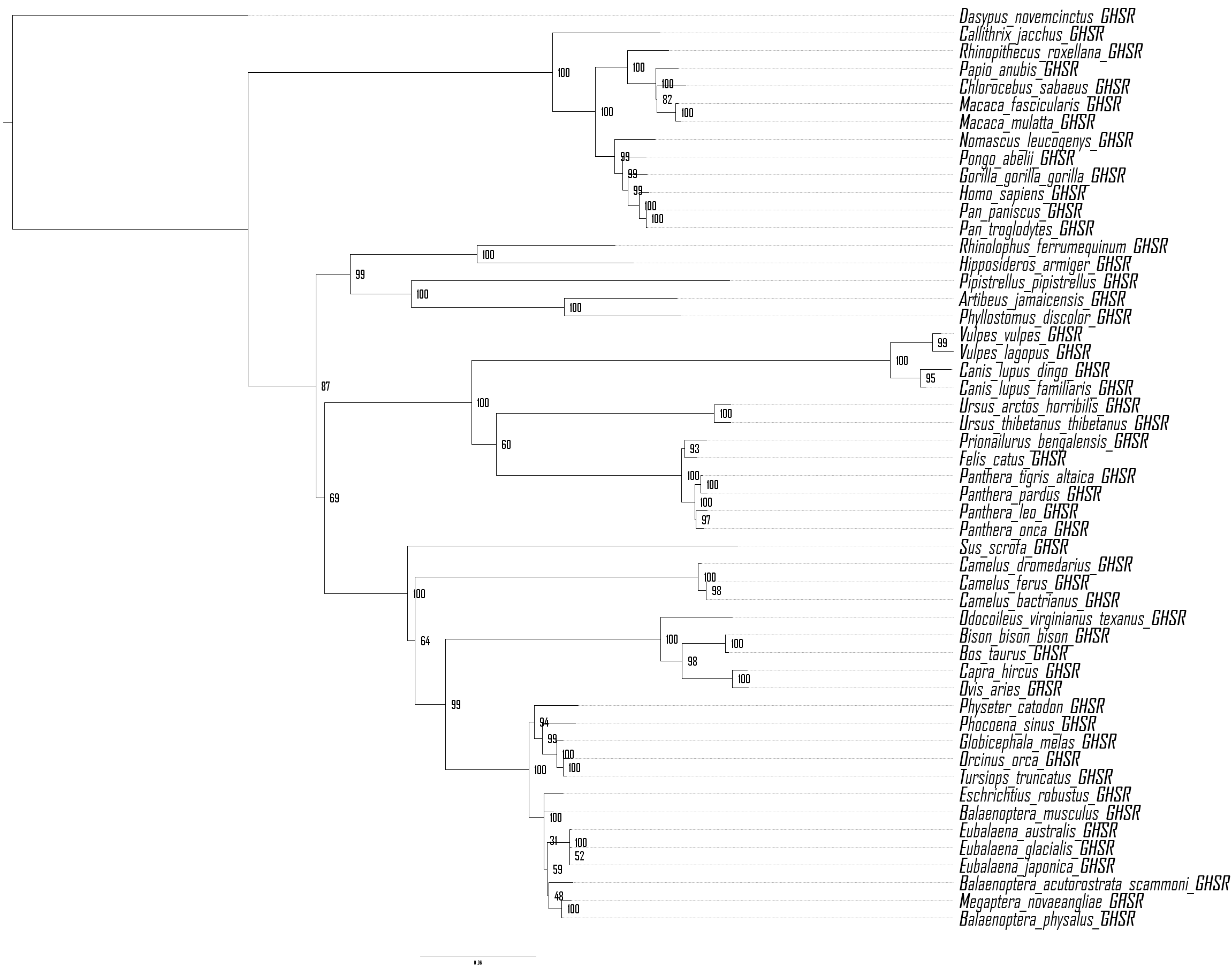


**Figure S2**: Maximum likelihood tree generated by IQ−TREE constructed from the promoter region of the GHSR gene. Numbers under nodes represent bootstrap support.


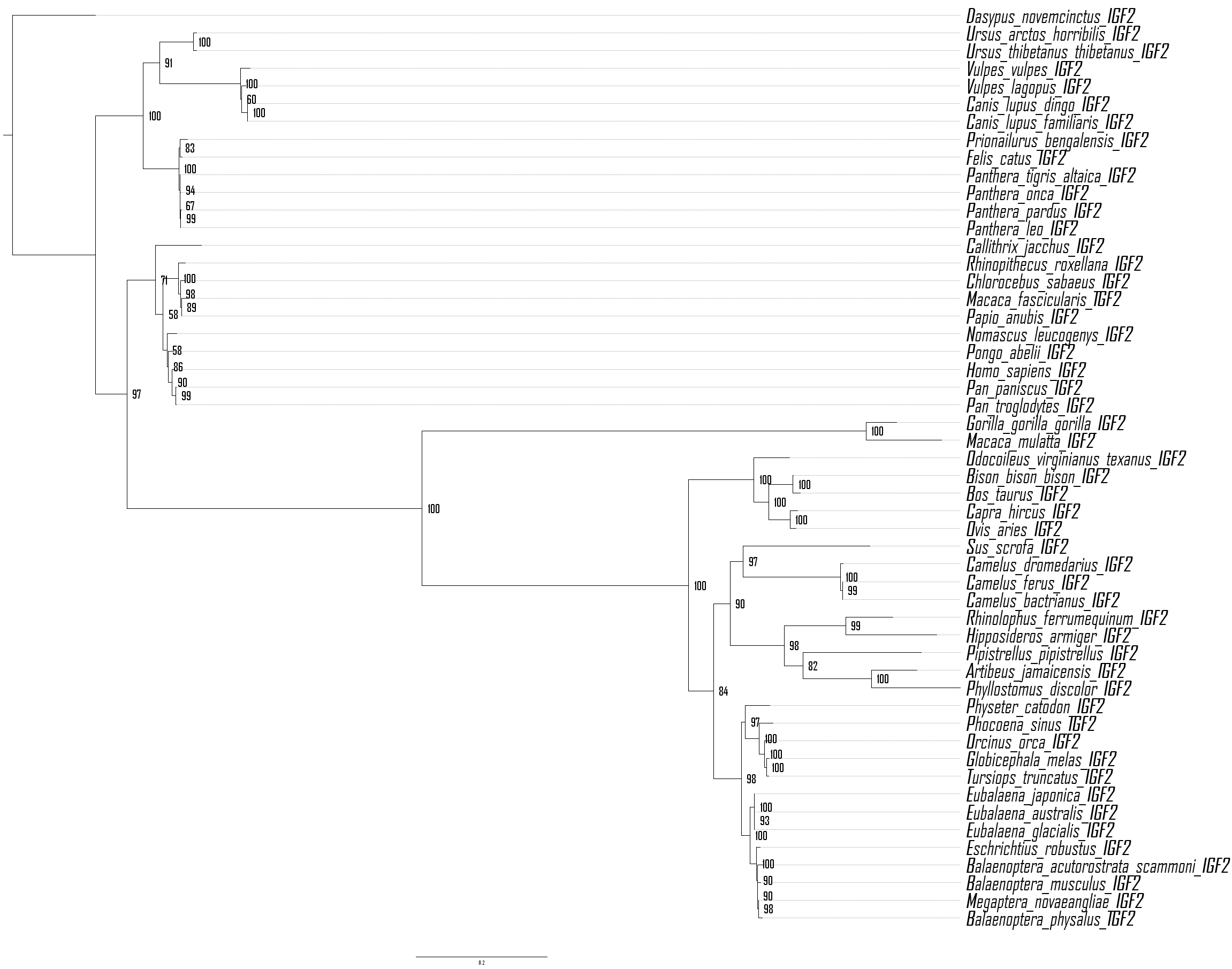


**Figure S3**: Maximum likelihood tree generated by IQ−TREE constructed from the promoter region of the IGF2 gene. Numbers under nodes represent bootstrap support.


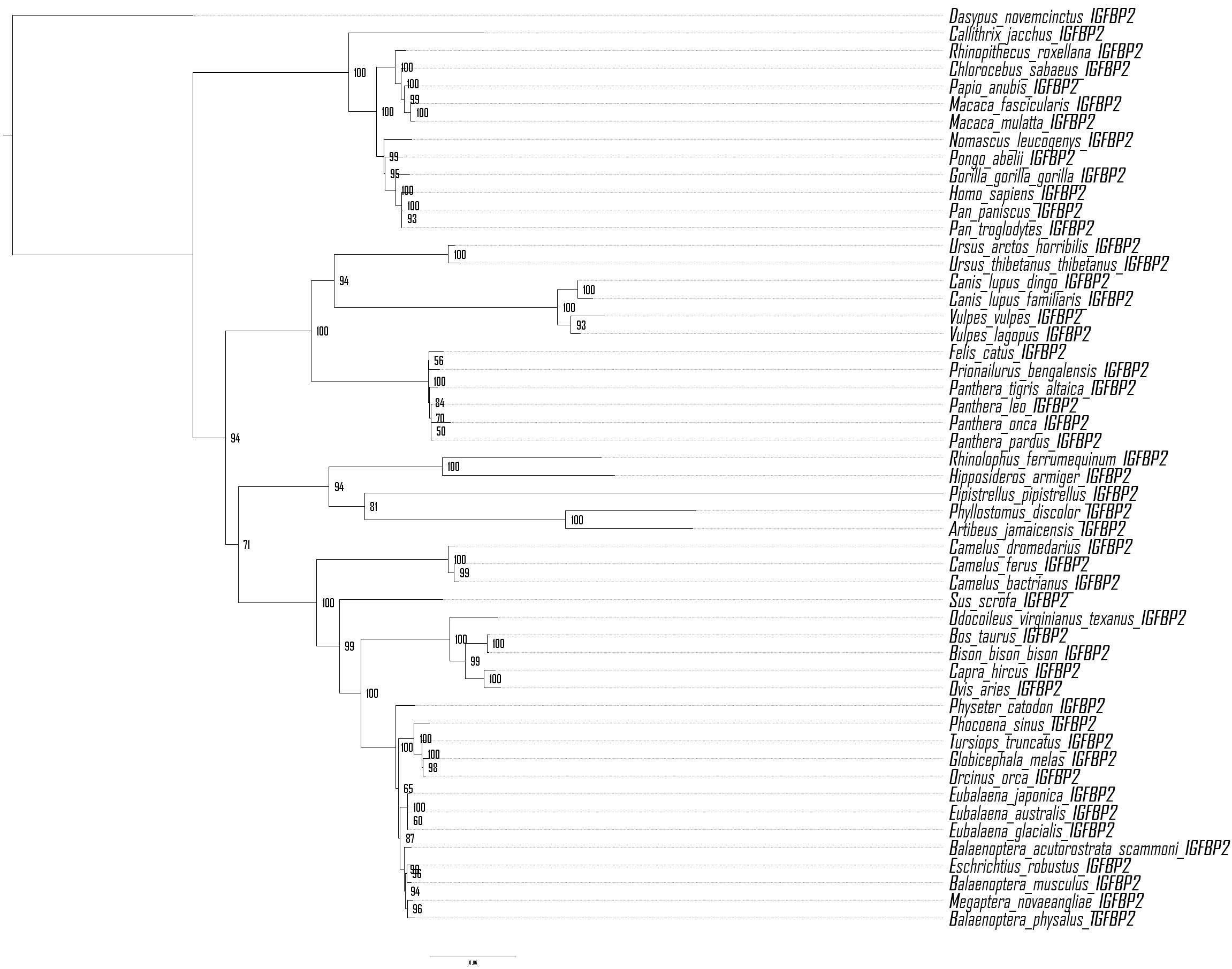


**Figure S4**: Maximum likelihood tree generated by IQ−TREE constructed from the promoter region of the IGFBP2 gene. Numbers under nodes represent bootstrap support.


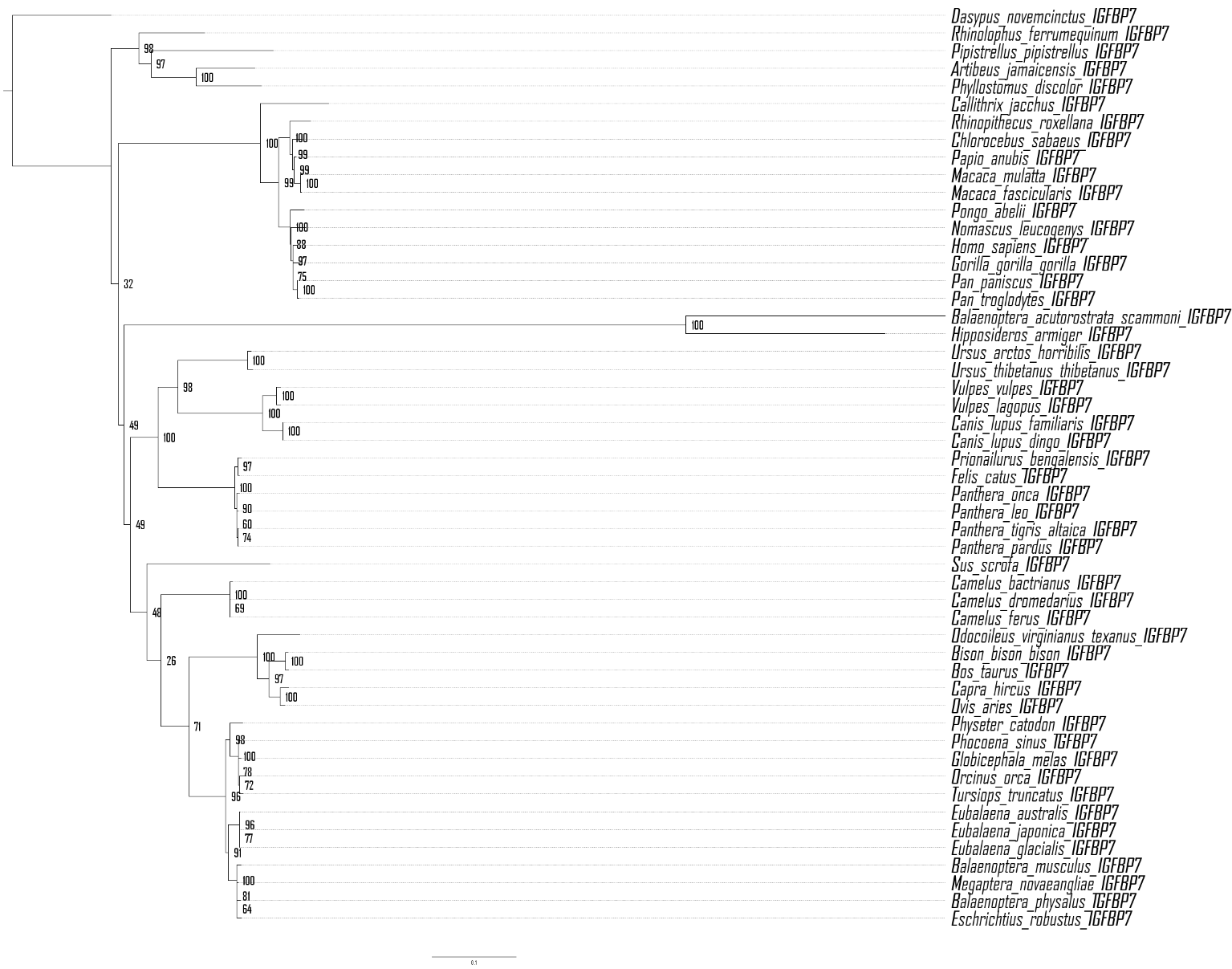


**Figure S5**: Maximum likelihood tree generated by IQ−TREE constructed from the promoter region of the IGFBP7 gene. Numbers under nodes represent bootstrap support.


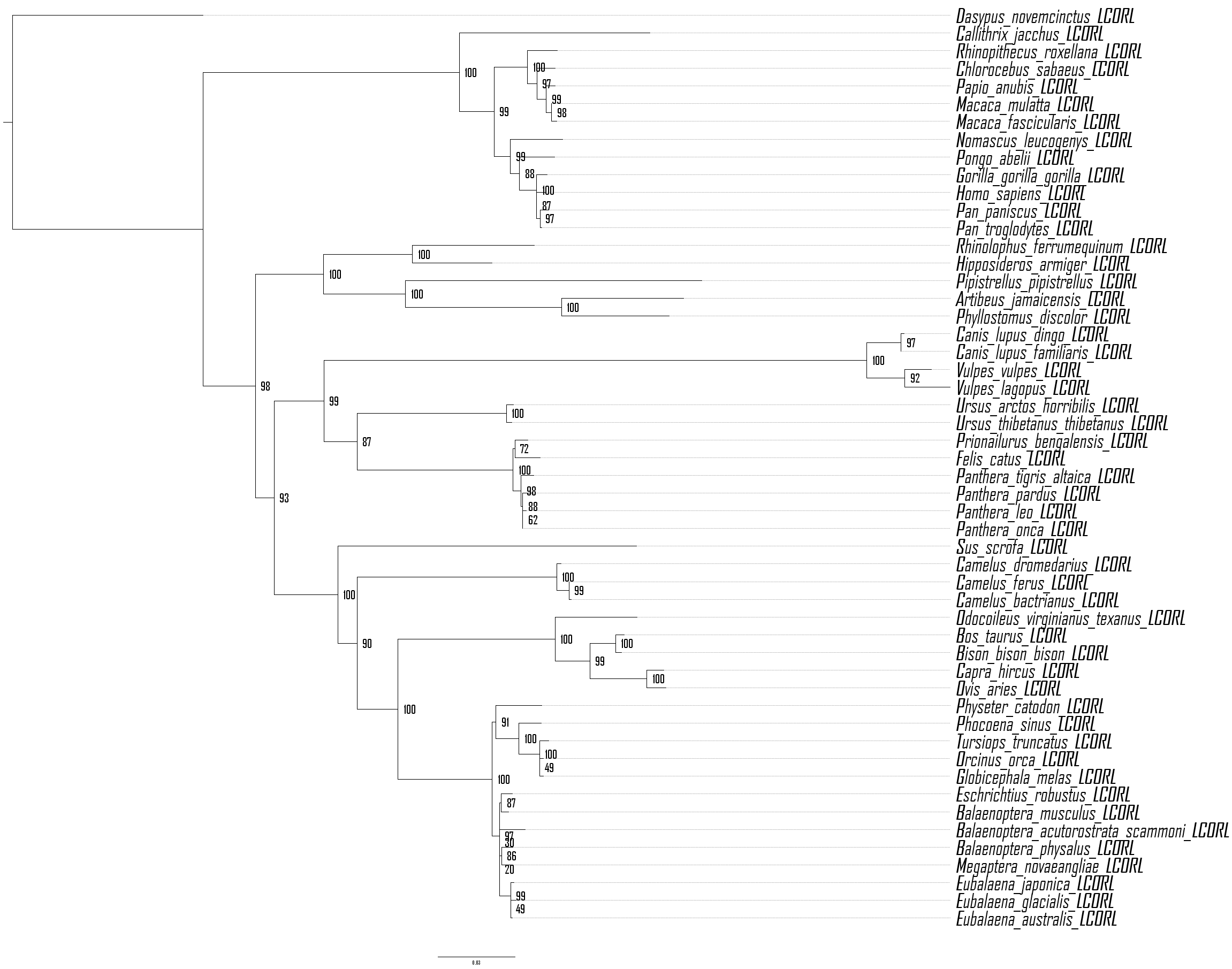


**Figure S6**: Maximum likelihood tree generated by IQ−TREE constructed from the promoter region of the LCORL gene. Numbers under nodes represent bootstrap support.


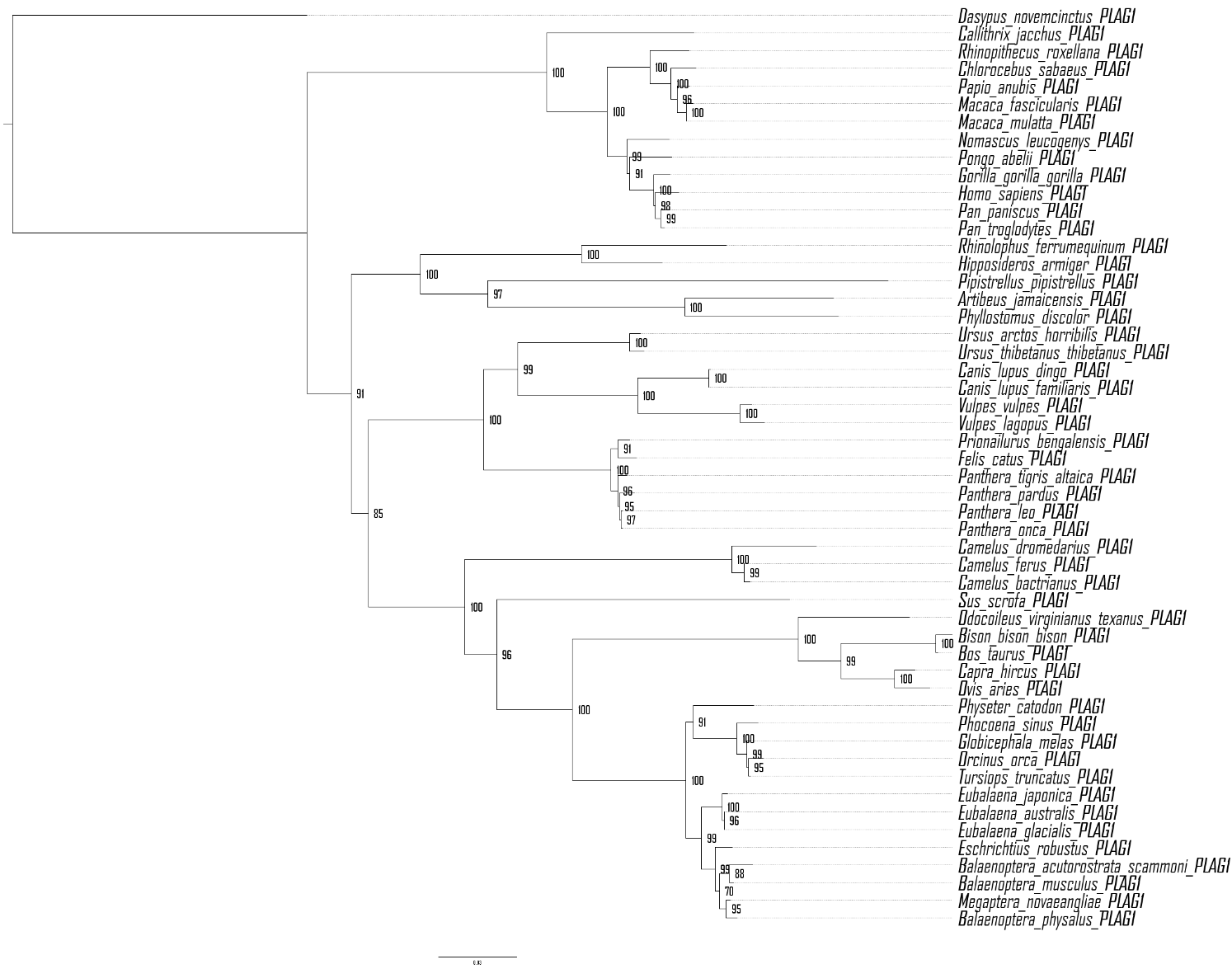


**Figure S7**: Maximum likelihood tree generated by IQ−TREE constructed from the promoter region of the PLAG1 gene. Numbers under nodes represent bootstrap support.


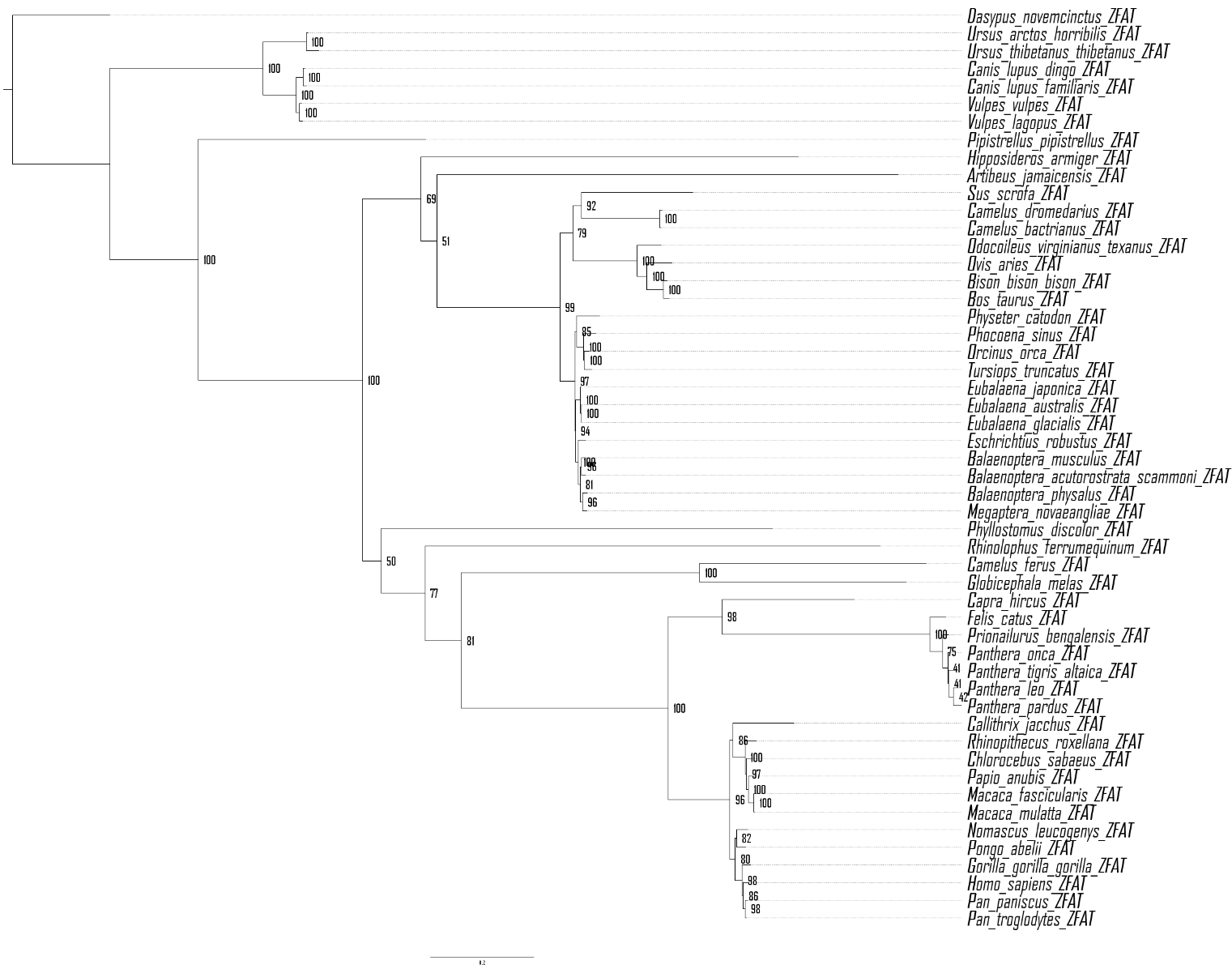


**Figure S8**: Maximum likelihood tree generated by IQ−TREE constructed from the promoter region of the ZFAT gene. Numbers under nodes represent bootstrap support.


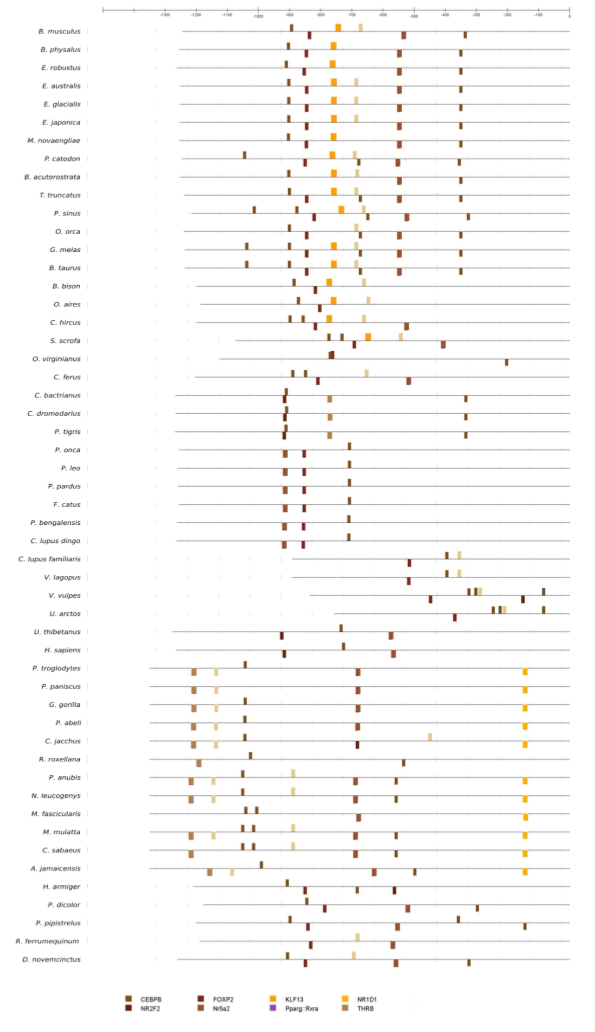


**Figure S9:** Enrichment pattern for the GHSR promoter implemented in the Ciiider program. The result shows transcription factors in bars. Mammals are cetaceans, artiodactyls, carnivores, primates, bats, and cingulates.


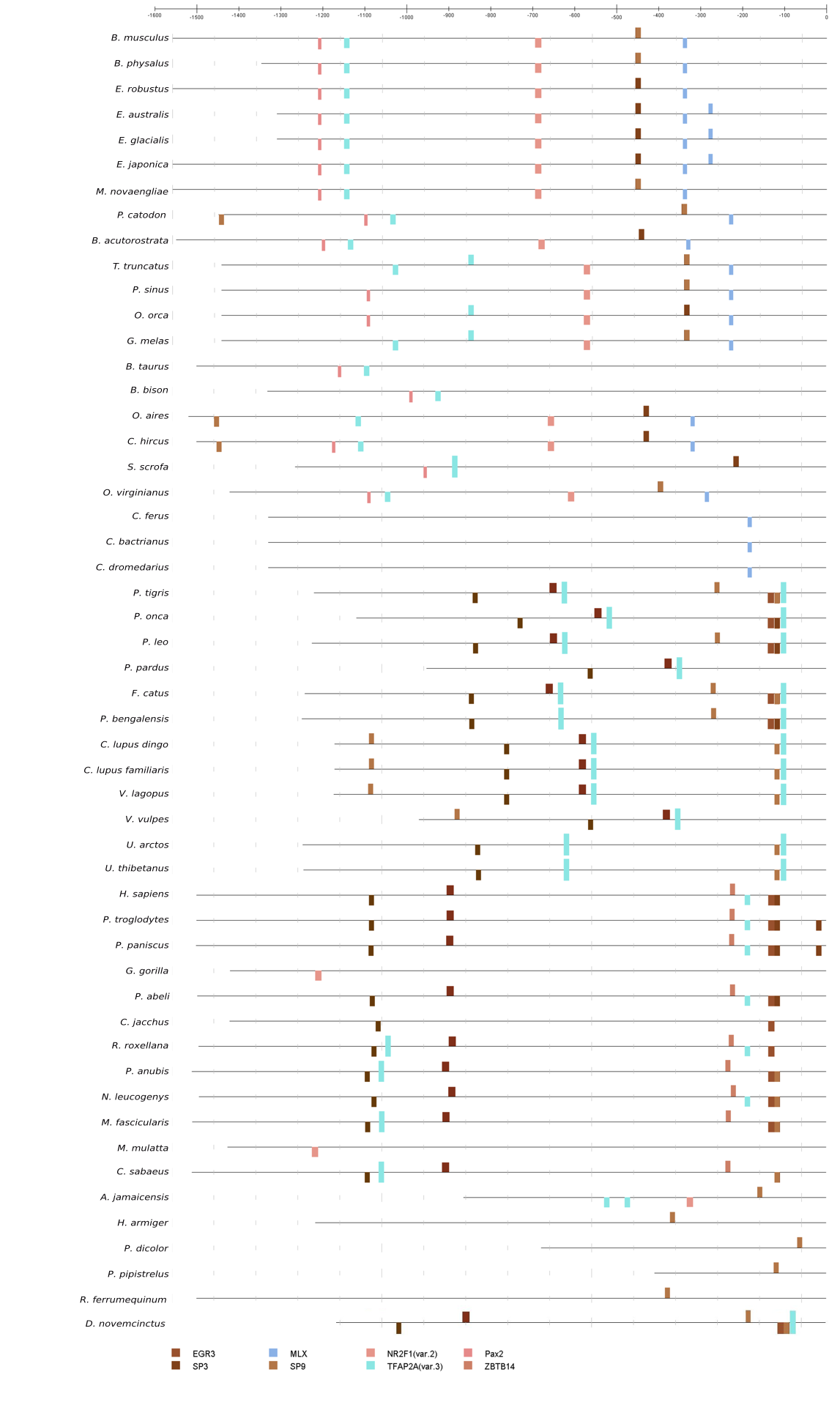


**Figure S10:** Enrichment pattern for the IGF2 promoter implemented in the Ciiider program. The result shows transcription factors in bars. Mammals are cetaceans, artiodactyls, carnivores, primates, bats, and cingulates.


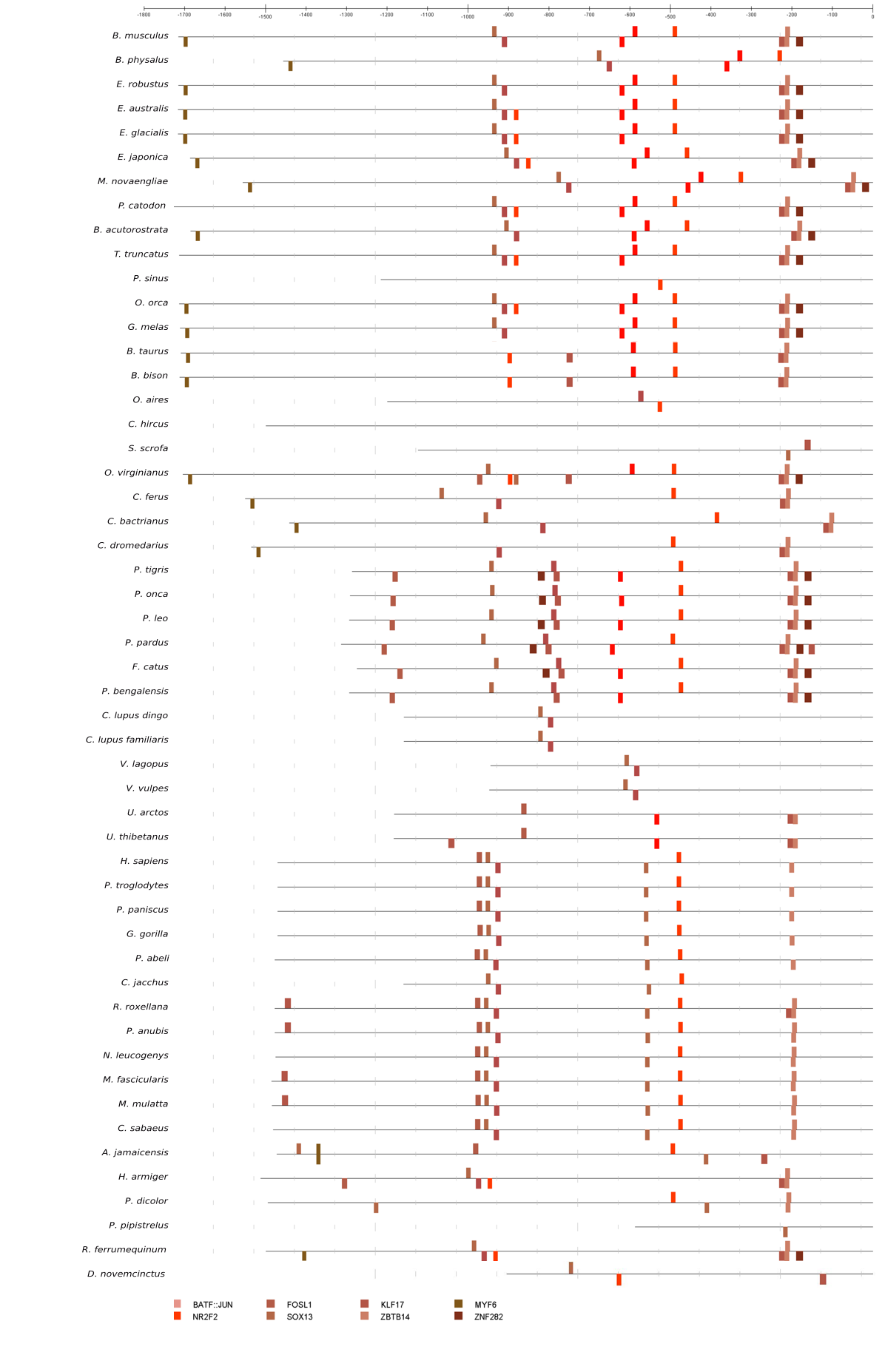


**Figure S11:** Enrichment pattern for the IGFBP2 promoter implemented in the Ciiider program. The result shows transcription factors in bars. Mammals are cetaceans, artiodactyls, carnivores, primates, bats, and cingulates.

**
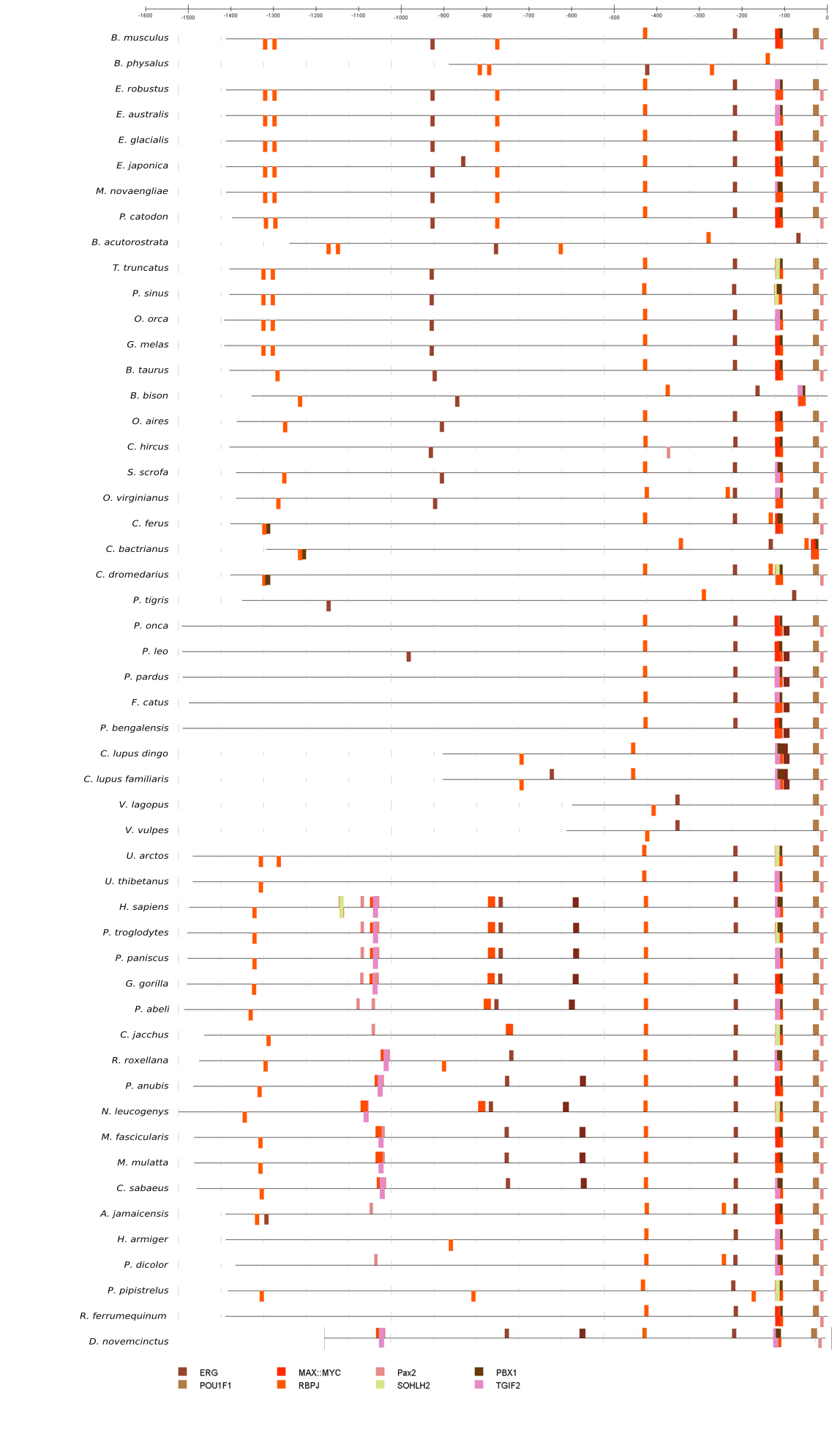
**

**Figure S12:** Enrichment pattern for the LCORL promoter implemented in the Ciiider program. The result shows transcription factors in bars. Mammals are cetaceans, artiodactyls, carnivores, primates, bats, and cingulates.

**
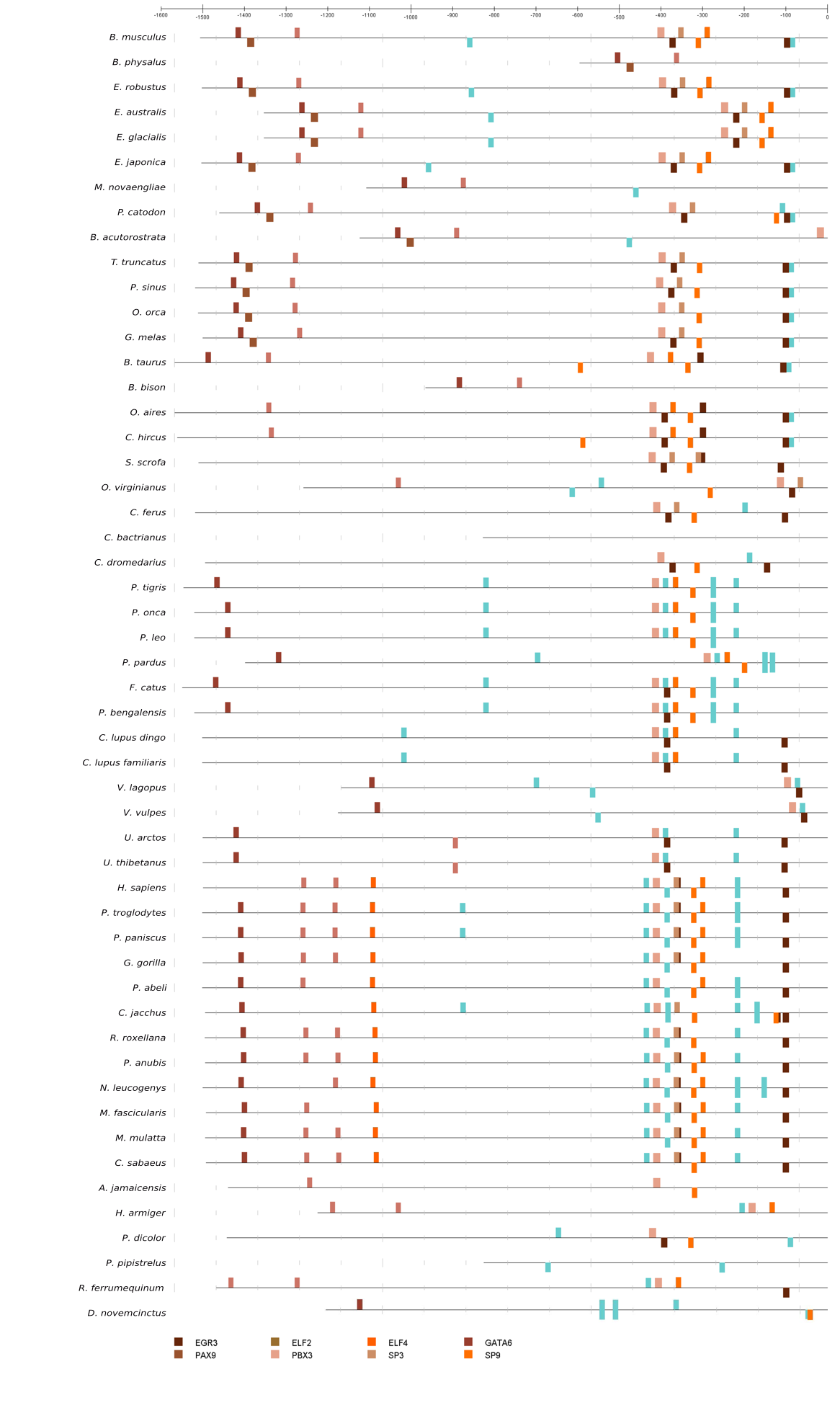
**

**Figure S13:** Enrichment pattern for the PLAG1 promoter implemented in the Ciiider program. The result shows transcription factors in bars. Mammals are cetaceans, artiodactyls, carnivores, primates, bats, and cingulates.


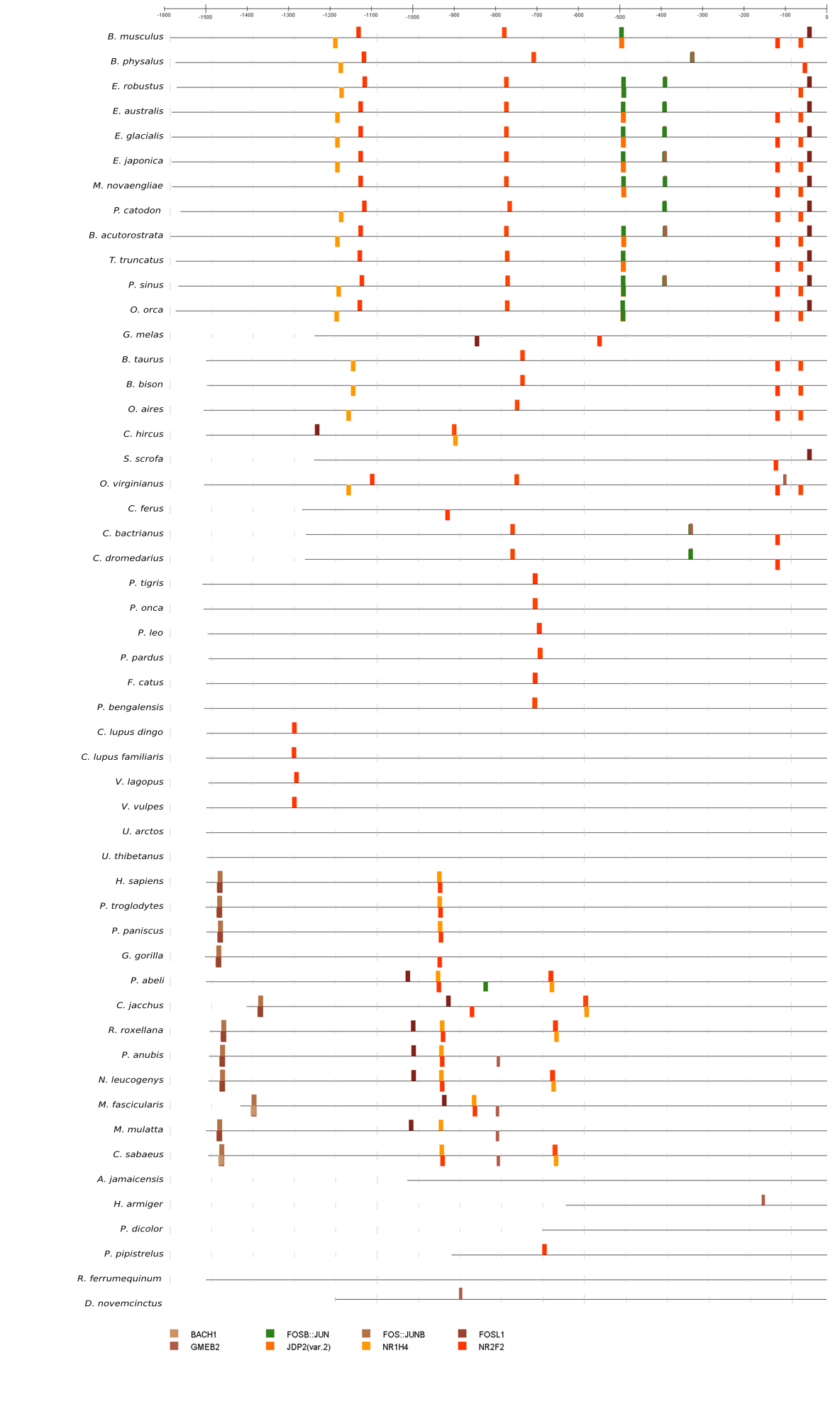


**Figure S14:** Enrichment pattern for the ZFAT promoter implemented in the Ciiider program. The result shows transcription factors in bars. Mammals are cetaceans, artiodactyls, carnivores, primates, bats, and cingulates.
